# Impact of a Manualized Multifocal Perinatal Home-Visiting Program Using Psychologists on Postnatal Depression: The CAPEDP Randomized Controlled Trial

**DOI:** 10.1371/journal.pone.0072216

**Published:** 2013-08-19

**Authors:** Romain Dugravier, Florence Tubach, Thomas Saias, Nicole Guedeney, Blandine Pasquet, Diane Purper-Ouakil, Susana Tereno, Bertrand Welniarz, Joana Matos, the CAPEDP study group, Antoine Guedeney, Tim Greacen

**Affiliations:** 1 Unité de périnatalité, Centre Hospitalier Sainte-Anne, Paris, France; INSERM U669 PSIGIAM, Paris, France; 2 AP-HP, Hôpital Bichat, Département d’Epidémiologie et Recherche Clinique, Paris, France; Université Paris Diderot, Sorbonne Paris Cité, Paris, France; INSERM, CIE801, Paris, France; 3 Laboratoire de Recherche, Etablissement Public de Santé Maison Blanche, Paris, France; Institut National de Prévention et d’Education pour la Santé, Saint-Denis, France; Département de Psychologie, Université du Québec à Montréal, Montréal, Canada; 4 Institut Mutualiste Montsouris, Paris, France; 5 AP-HP, Hôpital Bichat, Département d’Epidémiologie et Recherche Clinique, Paris, France; INSERM, CIE801, Paris, France; 6 CHRU Montpellier, Médecine Psychologique de l’Enfant, et de l’Adolescent (MPEA), Hôpital Saint Eloi, Montpellier, France; INSERM U894, Center for Psychiatry and Neurosciences, Team 1, Paris, France; 7 Institut de Psychologie, Université Paris Descartes, Sorbonne Paris Cité, Paris, France; Laboratoire de Psychopathologie et Processus de Santé (LPPS - EA4057), Paris, France; 8 Etablissement Public de Santé Ville Evrard, Neuilly-sur-Marne, France; 9 AP-HP, CHU Pitié-Salpêtrière, Paris, France; 10 AP-HP, Service de Pédopsychiatrie, Hôpital Bichat Claude-Bernard, Paris, France; INSERM U669 PSIGIAM, Paris, France; 11 Laboratoire de Recherche, Etablissement Public de Santé Maison Blanche, Paris, France; University of Pennsylvania, United States of America

## Abstract

**Context:**

Postnatal maternal depression (PND) is a significant risk factor for infant mental health. Although often targeted alongside other factors in perinatal home-visiting programs with vulnerable families, little impact on PND has been observed.

**Objective:**

This study evaluates the impact on PND symptomatology of a multifocal perinatal home-visiting intervention using psychologists in a sample of women presenting risk factors associated with infant mental health difficulties.

**Methods:**

440 primiparous women were recruited at their seventh month of pregnancy. All were future first-time mothers, under 26, with at least one of three additional psychosocial risk factors: low educational level, low income, or planning to raise the child without the father. The intervention consisted of intensive multifocal home visits through to the child’s second birthday. The control group received care as usual. PND symptomatology was assessed at baseline and three months after birth using the Edinburgh Postnatal Depression Scale (EPDS).

**Results:**

At three months postpartum, mean (SD) EPDS scores were 9.4 (5.4) for the control group and 8.6 (5.4) for the intervention group (p = 0.18). The difference between the mean EPDS scores was 0.85 (95% CI: 0.35; 1.34). The intervention group had significantly lower EPDS scores than controls in certain subgroups: women with few depressive symptoms at inclusion (EPDS <8): difference = 1.66 (95%CI: 0.17; 3.15), p = 0.05, adjusted for baseline EPDS score), women who were planning to raise the child with the child’s father: difference = 1.45 (95%CI: 0.27; 2.62), p = 0.04 (adjusted); women with a higher educational level: difference = 1.59 (95%CI: 0.50; 2.68) p = 0.05 (adjusted).

**Conclusion:**

CAPEDP failed to demonstrate an overall impact on PND. However, post-hoc analysis reveals the intervention was effective in terms of primary prevention and in subgroups of women without certain risk factors. Effective overall reduction of PND symptomatology for young, first-time mothers presenting additional psychosocial risk factors may require more tailored interventions.

**Trial registration:**

ClinicalTrials.gov NCT00392847

Promoting Parental Skills and Enhancing Attachment in Early Childhood (CAPEDP)

## Introduction

Postnatal maternal depression (PND) is strongly related to subsequent child mental health disorders, particularly insecure attachment [1] and cognitive, social and emotional developmental disorders during preschool years and beyond [2]. At the age of 11, children whose mothers suffered from PND are four times more likely to have a psychiatric disorder than children of non-depressed mothers [3], [4]. Furthermore, PND is not infrequent: from 10% to 15% of mothers present significant postnatal depression [5] and prevalence is even higher in women with a previous history of depression or with psychosocial risk factors such as low income or partner-related stress during pregnancy [6], [7].

Given this observed association between PND and infant mental health, it has come to be of major concern for home-visiting interventions, which often include it as a specific target alongside other mediating factors of child mental health. Most of these programs draw upon attachment theory [8], self-efficacy theory [9] and human ecological systems theory [10] as bases for their interventions targeting these mediating factors [11]. With regard to PND, in programs such as Healthy Families in America, Nurse-Family Partnership or Early Head Start, home-visiting teams work with mothers using standard mental health promotion strategies: developing ways of handling stress in difficult situations, teaching problem-solving skills that promote self-efficacy, providing practical assistance, and working with mothers to develop their social support networks [12]. However, few health promotion programs have demonstrated convincing results with regard to PND [13], [14], [15]. Only intensive interventions specifically targeting PND in women presenting risk factors for depression, beginning during pregnancy and continuing on through the postpartum period, seem to have some effect [16], [17].

The CAPEDP study *(Compétences parentales et Attachement dans la Petite Enfance: Diminution des risques lies aux troubles de santé mentale et Promotion de la résilience* - Parental Skills and Attachment in Early Childhood: reducing mental health risks and promoting resilience) was the first randomized, controlled trial assessing an evidence-based, home-visiting, infant mental health promotion program to take place in France [18]. The program had three primary objectives: improving child mental health at the age of two and, with regard to potential mediating variables for infant mental health, reducing PND at three months postpartum, and optimising the quality of the home environment when the children were 12 months old. The present article describes the impact of the CAPEDP intervention on PND when the children were three months old.

### The French Context

At the close of World War II, France established nation-wide, community-based, mother-child support and prevention services with no out-of-pocket payment, known as *Protection Maternelle et Infantile* (Mother and Child Protection Services or PMI). Today, mothers have direct access to PMI centres free of charge from the beginning of pregnancy right through to their child’s third birthday. More than one in five families with a new-born child receives home visits by nurses if identified by maternity ward staff as being potentially vulnerable. France was also the first Western country to develop free community mental health services in every neighbourhood for both adults and children. Each child and adolescent mental health service provides care with no out-of-pocket payment for a population area of approximately 250,000 inhabitants and, although with limited resources, teams are able to conduct home visits if deemed necessary for the child’s mental health or safety. Families also automatically access family benefits provided by local government to help raise their children, under the condition that they accept to bring their child in for a small number of health check-ups and compulsory vaccinations. With regard to mental health, although PMI nurses receive little specific training on mental health promotion or prevention and little organised psychological supervision, they can and do refer families directly to their local community child and adolescent mental health services.

## Materials and Methods

The protocol for this trial and supporting CONSORT checklist are available as supporting information; see Checklist S1 and Protocol S1.

### The CAPEDP intervention

The purpose of the CAPEDP study was to evaluate the effectiveness of a home-visiting program in the context of this generous French health and social care system and based on the latest evidence-based recommendations in terms of promoting infant mental health, reducing PND and promoting parenting skills [19]. The program targeted mothers presenting risk factors associated with greater likelihood of child mental health disorders.

The manualized intervention was specifically tailored to empower mothers in terms of developing parenting skills, using the health and social care system, and making the most of their personal networks and local community services. A team of home-visiting psychologists was specifically trained to promote mental health and attachment quality, provide social and emotional support within a solid working alliance, and address depression should it occur. All psychologists received weekly individual supervision with psychiatrists from the research team and monthly group supervision with the chief investigator, a Professor of Child Psychiatry.

Although perinatal psychopathology is a traditional part of all undergraduate training for psychologists in France, preventing maternal depression and managing it should it occur were specifically addressed in a half-day training module in the initial training program for the CAPEDP home-visiting team. Furthermore, the intervention manual proposed a series of discussion topics concerning perinatal depression to be raised during home visits in the prenatal period: recognizing, understanding and handling the symptoms of depression; understanding the importance of social support; and accessing care as soon as needed. During these early home visits, the future mothers were given an information sheet on understanding “baby blues” and what to do if they felt they were experiencing symptoms such as moodiness, sadness, difficulty sleeping, irritability, appetite changes or concentration problems. If a member of the home-visiting team identified depressive symptoms during any prenatal visit, they were instructed to bring the subject up with their supervisor, to be prepared to make additional home visits over and above those already programmed in the study protocol and, if necessary, to refer the participant to a community mental health center. During the postnatal period (0-3 months), the intervention manual reminded home visitors to pay particular attention to symptoms of maternal depression and to use active listening approaches with any mother presenting what might be initial symptoms of depression. If symptoms persisted or worsened, an individualized care protocol was developed in collaboration with the home-visitor’s supervisor.

A total of 14 home visits were scheduled for the period concerned by the present study: six times during the prenatal period beginning at the seventh month of pregnancy and eight times during the first three months of the child’s life.

### Study Design and Setting

The CAPEDP Study was a prospective, randomized controlled, multicentre trial with two parallel arms comparing the CAPEDP intervention to usual care, using Prospective Randomized Open Blinded Endpoint (PROBE) methodology [20], and with a 27-month follow-up for young primiparous women presenting risk factors associated with greater likelihood of subsequent child mental health disorders. Usual care involved access to the PMI and community mental health networks with no out-of-pocket payment, free antenatal maternity screenings, and a variety of social benefits, as described above. In addition, the intervention group received the CAPEDP home-visiting program.

The CAPEDP intervention and the methodology used to evaluate its impact are described elsewhere [18], [21] (articles provided as supplementary files for the reviewers). The study protocol was approved for all centres by the Institutional Review Board ‘Comité de Protection des Personnes Ile de France IV’ (2006/37). Written informed consent was obtained from all participants before integrating the study.

The trial is registered as ClinicalTrials.gov number *NCT0039284*

### Participants

As stated above, eligibility criteria limited participation to mothers in situations of medium to high vulnerability with regard to their future child’s mental health. All consecutive women consulting in the second trimester of pregnancy (from 12 to 27 weeks since first day of the last menstrual period (LMP)) in ten public maternity wards in Paris and its surrounding suburbs were assessed for eligibility. Women were eligible if they presented the following characteristics: living in the intervention area (Paris and its inner suburbs); fluent enough in French to give valid informed consent, benefit from the intervention and participate in assessment sessions; less than 27 weeks after LMP at their first assessment interview; and, as required by French law on clinical research, registered with the national health insurance scheme or its equivalent for non-French participants. In terms of risk factors for their future child’s mental health, they also had to be first-time mothers; less than 26 years old; and meet at least one of the following three criteria: 1) having less than twelve years of education, 2) planning to bring up their child without the child’s father, and 3) having low income, defined as being eligible for French national social welfare health insurance (*Couverture Maladie Universelle Complémentaire*) or, for undocumented migrants, Government Medical Aid (*Aide médicale d’Etat)*.

Exclusion criteria were: women who were going to be impossible to follow up (for example, women who were planning to move away from the Greater Paris Area after their child was born), women receiving social or medical care for reasons other than those listed in the above inclusion criteria (such as substance abuse, serious mental illness, or other chronic diseases requiring close follow-up), and women who did not consent to participate.

Participation in the study was proposed to eligible women in the waiting room of the maternity hospital, prior to a prenatal appointment. During this encounter or at a second appointment if she requested more time to make up her mind, the future participant signed the consent form.

### Randomisation and masking

After completing baseline screening and informed consent procedures, participants were randomly assigned in a 1∶1 ratio to either the CAPEDP intervention or the usual care group using a computer-generated randomisation sequence, stratified by recruitment centre, with random block sizes of 2, 4 or 6 participants. The Clinical Research Unit of Bichat Hospital, Paris, France, centrally generated this sequence. Assignment of participants was concealed using centralized randomisation through fax in the Clinical Research Unit. Investigators thus had no knowledge of the next assignment in the sequence in this open label trial. All investigators, all psychologists performing the CAPEDP intervention and all participants were blinded to assignment before, but not after, randomisation. However, in accordance with PROBE methodology, all outcome assessors were blinded to assignment and no investigators, intervention psychologists or participants had any knowledge of aggregate outcomes at any point during the course of the study.

### Data Collection and Definitions

Assessments were conducted during specific home visits by a team of four trained psychologists, working independently from the psychologists performing the CAPEDP intervention and with no knowledge of whether the women being evaluated were in the intervention group or the control group. Assessment took place at baseline for demographic and health characteristics and then at 3, 6, 12, 18 and 24 months after the child’s birth for both groups. The assessment team received specific training on each of the assessment instruments. Individual and group supervision was provided for all members of the assessment team, to support them in handling difficult situations during evaluation, for example in situations of abuse or neglect, developmental delay, suicidal ideation in the mother or serious social problems. Whenever necessary, families were addressed to social or medical services.

### The Edinburgh Postnatal Depression Scale (EPDS)

Level of depressive symptoms was assessed for all women included in the study using the Edinburgh Postnatal Depression Scale (EPDS) [22]. The French version of the EPDS has been validated in the French population for both postnatal [22] and prenatal use [23]. The EPDS is a 10-item, self-administered questionnaire specifically developed for use during the postnatal period but also validated and commonly used for assessing prenatal depression. The questions focus on the psychological rather than the somatic aspects of depression. Mothers respond to items on a 4-point Likert scale. Total scores range from 0 to 30. Higher scores indicate higher levels of depressive symptoms.

Although a high EPDS score does not in itself confirm a diagnosis of depression, scores above a certain cut-off point indicate a probable depressive disorder. In the present study, the EPDS was administered at inclusion before the third trimester of pregnancy and at three months postpartum. In the French population, a cut-off score of >10 has been found to be optimal for postnatal screening for depression in mothers evaluated by nurses in PMI centers [22]. Concerning prenatal depression, a cut-off score of >11 has been shown to be appropriate [23]. Finally, a score of < 8 has been identified to define ‘low symptomatology’ [24].

### Population characteristics

At baseline and, when appropriate, at follow-up visits, the following data were collected: demographic data included age, sex, marital status, ethnicity, family characteristics, household composition, characteristics of the partner and, if different, the father of the coming child, whether the pregnancy was planned or not, number of years of schooling, educational level attained, employment status, and income; health variables included PND, self-perceived state of health, and use of tobacco, alcohol or drugs.

### Statistical Analysis

The trial was designed to establish whether the CAPEDP intervention was superior to usual care in terms of preventing PND as assessed using the EPDS, the quality of the home environment assessed with the Home Observation for the Measurement of the Environment (HOME) at the age of one, and child psychopathology at the age of two, assessed using the Child Behaviour Checklist (CBCL 1½–5).

To account for possible attrition and have sufficient power to answer all three primary objectives, the project needed to recruit 440 families. This was ample with regard to PND: indeed, assuming a mean of 12.1 on the EPDS (SD 4.6) for the usual care group [25], 113 participants per study group would have sufficed to detect a 2-point difference in the EPDS score with 90*%* power and a 2*-*sided significance level of *α  =  5%*. The data were summarized using means and standard deviations for continuous data, and frequencies and percentages for categorical data. Analyses were conducted according to the modified intention-to-treat principle: all participants were taken into account within their particular assignment group, whatever might have happened during the study, and all randomized participants who had at least one assessment visit within the first year of the study (including the baseline visit) were included for analysis.

Missing data for EPDS at three months postpartum were handled using multiple imputation for women who had at least one assessment visit during the 12 months after inclusion (modified ITT). For mothers who had no evaluation visit during the first year, imputation concerning data at three months postpartum was not possible (no baseline data except eligibility criteria). They were therefore not included in the present analysis. Although analysis of the eligibility criteria revealed no apparent difference with regard to the other women included in the modified ITT analysis, the benefits of randomization may well have been compromised to some extent. For this reason, due to the fact that mean EPDS scores differed between the two groups at baseline, both primary and secondary analyses were adjusted for baseline EPDS scores using linear regression. All in all, data concerning 183 women from the control group and 184 women from the intervention group were involved in the final analyses.

For the primary analysis, the between-group absolute difference in the EPDS score at three months was analyzed using Student’s t test. For the secondary analyses, data that were normally distributed were compared between groups using Student’s t test for continuous data and the chi-square test for categorical data. If not, appropriate non-parametric methods were used.

In the primary analysis, subgroup analyses were pre-specified for each of the three additional risk factors that had been chosen as inclusion criteria (planning to raise child without the father; low income level; low educational level), as well as the number of these additional risk factors at inclusion. Further post-hoc subgroup analyses were conducted for risk and protective factors collected during the first assessment interview at the future mother’s home: prenatal depression (EPDS >11); low level of depressive symptoms (EPDS <8); unplanned pregnancy; considers herself to be poor; first generation immigrant; tobacco, alcohol or drug use during pregnancy; separation of the future mother’s parents before she was 11 years old; death of one or both parents before she was 11 years old. The impact of the intervention on PND symptomatology at three months postpartum within each of these subgroups was analyzed using the same methods as for the main analysis. As EPDS scores differed between the two groups at baseline, the analysis was adjusted for baseline EPDS score using linear regression.

Baseline factors related to attrition when the children were three months old were investigated using Student and Chi-square tests for continuous and categorical variables respectively.

In the intervention group, the proportion of home visits that actually took place with regard to those scheduled was used to describe compliance to the CAPEDP program.

All statistical analyses were deemed significant at a 5% confidence level using two-sided tests. Statistical analyses were performed using SAS software version 9.2 (SAS Institute Inc., Cary, NC).

Data are available on request from the corresponding author.

## Results

**Figure 1:**
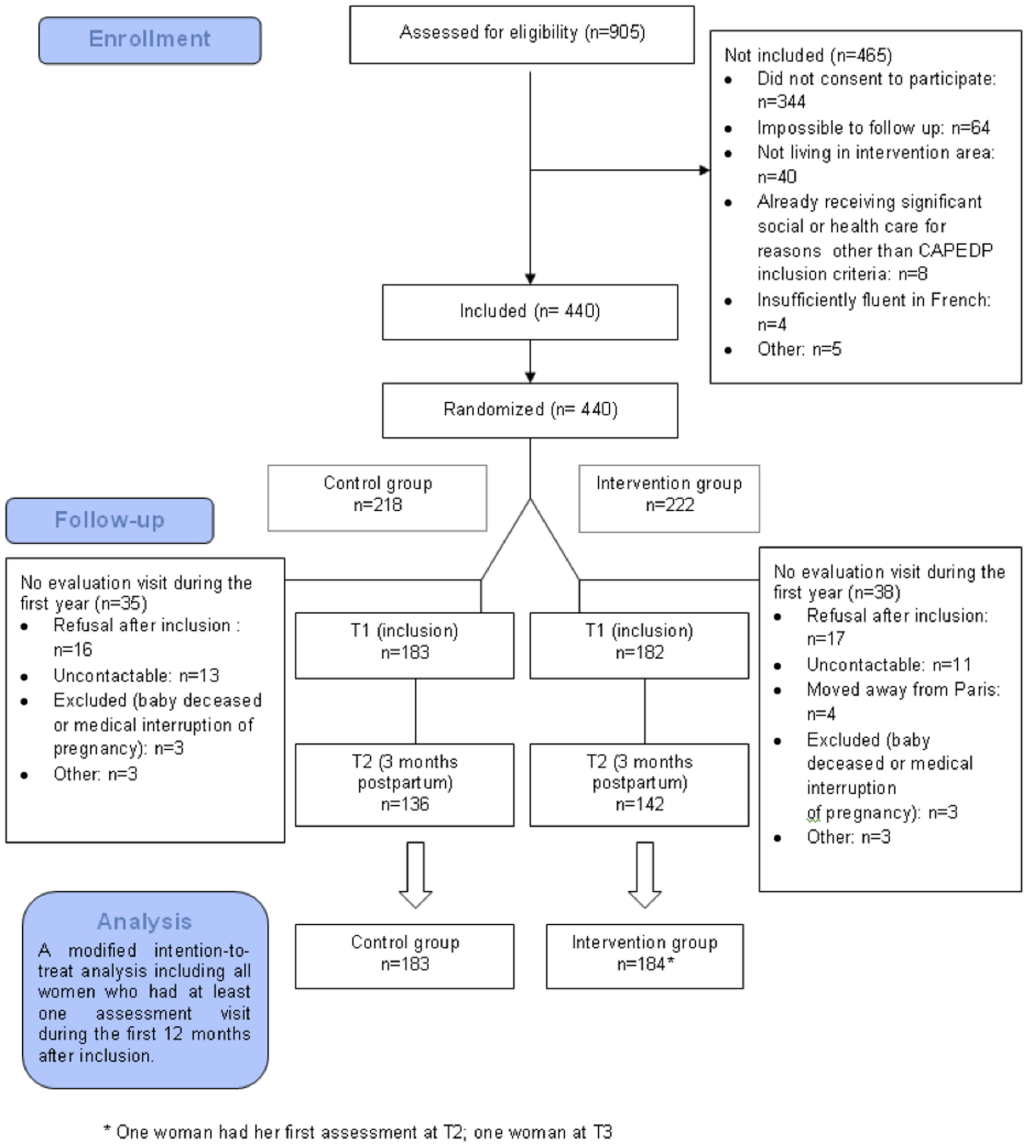
CAPEDP Flowchart Diagram.

A total of 440 women accepted to participate in the study and signed the consent form in the maternity ward at the prenatal visit. Of these, 73 (16.6% of the initial 440) then proceeded to withdraw their consent or could not be contacted during the first year of the study. These women were therefore not included in the modified intent to treat population (see Figure 1: flowchart diagram) because no data were available for them apart from initial eligibility criteria. At the second evaluation point, when the children were three months old, a further 88 mothers could not be interviewed, i.e. an additional 20% of the total initial sample. Comparisons between the 277 who presented at this interview and the 88 who did not, revealed that those who presented were more likely to have only one of the three additional risk factors at inclusion rather than having two or all three (p = 0.0165). Among women with only one additional risk factor at inclusion (apart from being first-time mothers and young), only 18.6% left the study before the third month of postpartum, whereas 29.4% did so in the group with two or three additional risk factors. No other inclusion variable and no variable collected at the first prenatal assessment interview, including depression as measured using the EPDS, was significantly associated with attrition at this second evaluation point.

The mean age of the 367 mothers at inclusion was 22.3. With regard to inclusion criteria risk factors, 307 (83.9%) had less than 12 years of school education (and 16.7% had less than 9 years); 170 (46.8%) had sufficiently low income to be eligible for government medical aid (CMU or AME); and 99 (27.1%) declared they were planning to bring up their child without the father. At the first assessment interview during pregnancy, 34.9% considered themselves to be poor; 44.2% were single; 37.9% had not planned their current pregnancy; 40.9% had been pregnant at least once before but their previous pregnancies had been interrupted; 52.1% were first generation immigrants; for 32.7% of the women, their parents had separated or divorced before the women were 11 years old, and for 11.6% of the women, one parent had died before the women were 11 years old (Table 1). The mean (SD) prenatal EPDS score was 11.1 (5.6) in the control group and 10.5 (5.6) in the intervention group; the difference between these mean EPDS scores was 0.63, 95%CI (0.12; 1.14), (p = 0.28).

**Table 1:** Demographic and health characteristics of the study population (N = 367).

		Total (N = 367)	Control group (N = 183)	Intervention group (N = 184)	p-value
**Inclusion Criteria Vulnerability Factors**					
Age	Mean (std)	22.3 (2.4)	22.2 (2.4)	22.5 (2.4)	(S) p = 0.2103
MD* = 0 (0.0%)					
Plans to raise her child without the father	Yes	99 (27.1%)	45 (24.7%)	54 (29.5%)	(K) p = 0.3041
MD = 2 (0.5%)	No	266 (72.9%)	137 (75.3%)	129 (70.5%)	
Low income (CMU/AME) **	Yes	170 (46.8%)	87 (48.3%)	83 (45.4%)	(K) p = 0.5697
MD = 4 (1.1%)	No	193 (53.2%)	93 (51.7%)	100 (54.6%)	
Low educational level (<12 years)	Yes	307 (83.9%)	154 (84.2%)	153 (83.6%)	(K) p = 0.8870
MD = 1 (0.3%)	No	59 (16.1%)	29 (15.8%)	30 (16.4%)	
Number of additional risk factors ***	1	189 (52.5%)	93 (52.0%)	96 (53.0%)	(K) p = 0.8369
MD = 7 (1.9%)	2 or 3	171 (47.5%)	86 (48.0%)	85 (47.0%)	
**Demographic and Health Factors**					
Living situation	Couple	203 (55.8%)	104 (56.8%)	99 (54.7%)	(K) p = 0.6818
MD = 3 (0.8%)	Single	161 (44.2%)	79 (43.2%)	82 (45.3%)	
Perceives herself to be poor	Yes	118 (34.9%)	58 (34.7%)	60 (35.1%)	(K) p = 0.9451
MD = 29 (7.9%)	No	220 (65.1%)	109 (65.3%)	111 (64.9%)	
French citizenship	Yes	211 (57.8%)	107 (58.5%)	104 (57.1%)	(K) p = 0.7974
MD = 2 (0.5%)	No	154 (42.2%)	76 (41.5%)	78 (42.9%)	
Planned pregnancy	Yes	226 (62.1%)	111 (60.7%)	115 (63.5%)	(K) p = 0.5712
MD = 3 (0.8%)	No	138 (37.9%)	72 (39.3%)	66 (36.5%)	
At least one prior interrupted pregnancy	Yes	149 (40.9%)	74 (40.4%)	75 (41.4%)	(K) p = 0.8463
MD = 3 (0.8%)	No	215 (59.1%)	109 (59.6%)	106 (58.6%)	
Educational level > 9 years	Yes	304 (83.3%)	147 (80.3%)	157 (86.3%)	(K) p = 0.1286
MD = 2 (0.5%)	No	61 (16.7%)	36 (19.7%)	25 (13.7%)	
First generation immigrant	Yes	190 (52.1%)	96 (52.5%)	94 (51.6%)	(K) p = 0.8768
MD = 2 (0.5%)	No	175 (47.9%)	87 (47.5%)	88 (48.4%)	
Tobacco, alcohol or drug use during pregnancy	Yes	94 (25.8%)	42 (23.0%)	52 (28.7%)	(K) p = 0.2079
MD = 3 (0.8%)	No	270 (74.2%)	141 (77.0%)	129 (71.3%)	
Separation of parents before age 11	Yes	111 (32.7%)	53 (32.1%)	58 (33.3%)	(K) p = 0.8121
MD = 28 (7.6%)	No	228 (67.3%)	112 (67.9%)	116 (66.7%)	
Death of a parent before age 11	Yes	36 (11.6%)	20 (12.4%)	16 (10.7%)	(K) p = 0.6287
MD = 56 (15.3%)	No	275 (88.4%)	141 (87.6%)	134 (89.3%)	

* MD: Missing Data.

** CMU (Couverture Maladie Universelle)/AME (Aide Médicale d’Etat).

*** Additional risk factors: plans to raise child without child’s father; low income; low educational level.

At three months postpartum, mean scores were 9.4 (5.4) for the control group and 8.6 (5.4) for the intervention group; the difference between these mean EPDS scores was 0.85, 95% CI (0.35;1.34); (crude comparison: p = 0.18; comparison adjusted for prenatal EPDS score: p = 0.33) (Table 2).

**Table 2:** Mean (standard deviation) EPDS scores during third trimester of pregnancy (baseline) and 3 months after birth.

		TOTAL (N = 367)	Control group (N = 183)	Intervention group (N = 184)	p-value
	N	367	183	184	
EPDS during 3^rd^ trimester of pregnancy	Mean (std)	10.8 (5.6)	11.1 (5.6)	10.5 (5.6)	(S) p = 0.28
EPDS at 3 months postpartum	Mean (std)	9.0 (5.4)	9.4 (5.4)	8.6 (5.4)	(S) p = 0.18 p = 0.33*

* p-value adjusted for prenatal EPDS score.

At baseline, 164 (44.7%) women had a prepartum EPDS score >11, 86 of whom were in the control group (47.0%) and 78 in the intervention group (42.4%). At three months postpartum, 69 mothers in the control group (37.7%) and 65 in the intervention group (35.3%) had a postpartum score >10 (p = 0.64).

**Figure 2:**
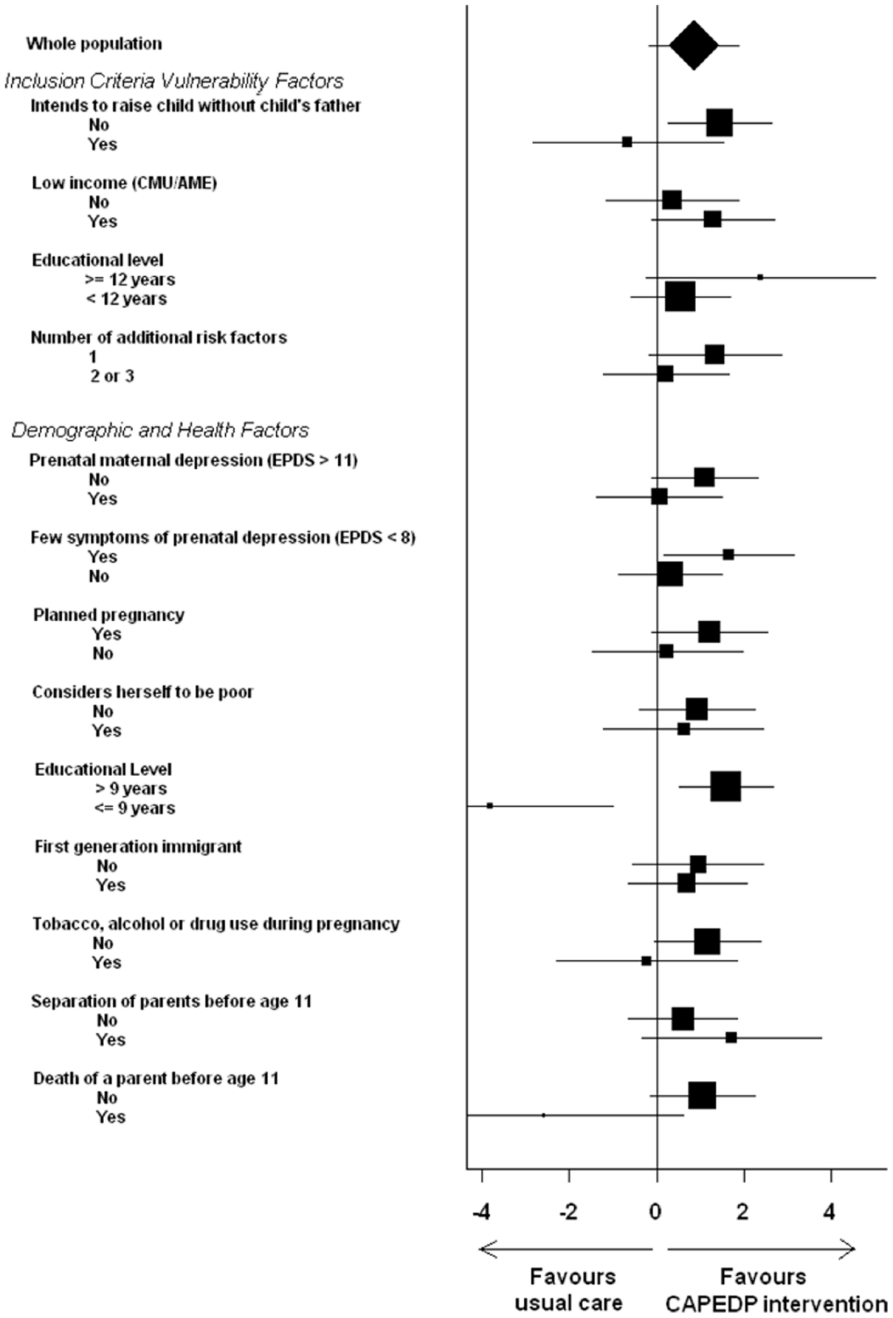
Absolute difference in EPDS score at 3 months postpartum between trial arms in subgroups of women sharing particular characteristics. Boxes indicate absolute differences. Box size is proportional to the number of patients involved in each sub-group. Horizontal lines indicate 95% confidence intervals.

However, in terms of primary prevention, among women with few self-declared depressive symptoms at inclusion (EPDS < 8), the difference between the mean EPDS scores (95% CI) of the control and the intervention group at three months postpartum was 1.66 (0.17; 3.15) (crude comparison: p = 0.18; comparison adjusted for prenatal EPDS score: p = 0.05) The intervention was also effective in terms of reducing PND at three months postpartum for the 266 mothers who, at their inclusion into the study, had declared that they were planning to bring up their child with the child’s father (difference between the mean EDPDS scores (95% CI) at three months postpartum: 1.45 (0.27; 2.62), p = 0.02 without adjustment for EPDS at inclusion, p = 0.04 with adjustment) and also for the 304 who had had more than nine years of education (difference between the mean EPDS scores (95% CI) at three months postpartum: 1.59 (0.50; 2.68), crude comparison: p = 0.004, comparison adjusted for prenatal EPDS score: p = 0.05 ). Similar tendencies, although not reaching statistical significance, were observed for women with various other vulnerability factors identified at the first assessment visit (see Figure 2).

Finally, within the intervention group, of the 6 planned prenatal home visits, an average (SD) of 3.2 (2.0) visits actually took place; in the first three months postpartum, mothers received 3.7 (2.6) of the 8 planned visits. Mothers with higher EPDS scores at three months postpartum tended to have received a greater number of home visits than less depressed mothers (p = 0.053; p adjusted for EPDS at baseline  =  0.067).

## Discussion

The current home-visiting intervention in an at-risk population in the Greater Paris Area was conducted by psychologists who had received specific trained to promote perinatal mental and physical health, identify depressive symptoms, provide support and refer to care if necessary. The intervention had no overall effect on the prevalence of PND symptomatology at three months postpartum. These results confirm findings from other studies, where multifocal interventions targeting infant mental health in vulnerable families have not generally proved to be effective with regard to reducing PND: Early Head Start [26], Healthy Families in America [27], [28], Nurse Family Partnership [19], Parents As Teachers [29], Healthy Families Alaska [30], Hawaii Healthy Start Program [31] and Healthy Families New York [32]. All these studies have in common the fact that they address at-risk populations with the general aim of improving infant mental health outcomes by targeting a number of determinants of infant mental health, amongst which PND.

In the present study, although levels of both prenatal and postnatal depressive symptoms evaluated with the EPDS are high (44.7% and 36.5% respectively), these results are comparable to those of other interventions in high-risk populations. In the Early Head Start program, 58% of mothers were depressed when included into the program just after their child was born [33]; a 1995 study in Akron, Ohio, found a 24.5% prevalence rate during pregnancy and 23.4% at postpartum in poor, inner-city mothers [34].

None of the above studies, however, including those integrating mental health training for home-visiting nurses or professional psychological support for depressed mothers, have demonstrated significant overall effects on PND [35]. Only interventions specifically targeting PND have shown promising results with at risk populations [16], [17], although these results have not been further replicated. Indeed, the only interventions that have proved successful in at-risk families are those specifically addressing only PND and focusing on providing care for existing depression, rather than aiming to prevent it [16], [36]. In the present study, post-hoc analysis reveals that the CAPEDP intervention was however effective in reducing levels of postpartum depression for women who, at baseline, had few depressive symptoms (EPDS<8), for those who declared they were planning to bring the child up with the child’s father (73.9% of the study population) and for those who had had more than nine years of education. Similarly a global tendency to efficacy was observed for women who, over and above the shared inclusion criteria of being first-time mothers and young, presented only one of the three additional inclusion criteria risk factors (low educational level, low income, or planning to bring up the child without the child’s father) rather than having two or all three, although it would be hazardous to jump to conclusions on this latter question given that women with more than one of the three additional risk factors at inclusion were also significantly more likely to have already dropped out of the study when PND symptomatology was measured at three months postpartum.

The current results, and particularly the lack of effect with regard to PND symptomatology in the overall sample, cannot however be interpreted without taking into account the specificity of the generous French social and health care system. The control group cannot be considered to have received no intervention: all subjects, including those in the care as usual group, had, in principle, easy access to both PMI and community mental health centers in their local neighborhoods. This is further compounded by a probable Hawthorne effect: women in the control group all had up to two home visits by professional psychologists for assessment purposes. If these women were discovered to be seriously depressed or if they requested help during evaluation, staff systematically referred them to care. The evaluation process may well have been an intervention in itself.

### Limits of the study

Attrition is a major challenge for long-term health promotion programs in general and, as mentioned above, this remains true for perinatal home-visiting programs [27]. In the Healthy Families New York program, one year after baseline, 50% of the mothers assigned to the intervention group had dropped out of the program; at the children’s second birthday, only one in three participants remained in the program [37]. Similar results were found in the Healthy Start program in Hawaii [12]. In the present study, attrition at three months postpartum, six months into the program, was also high (36.8%). No individual risk factor, from prenatal depression through to any socioeconomic factor, was associated with drop-out. However, the fact that future mothers who presented a greater number of risk factors for later infant mental health problems were significantly more likely to drop out of the program, with comparable drop-out rates in the control group and the intervention group, cannot be neglected.

A second limitation of the present study was that, although the home-visiting team, all qualified psychologists, had received specific instructions in this manualized intervention to work on questions of mood and depression as of day one, a qualitative analysis of their home-visiting case-notes showed little evidence of this theme actually being addressed as a priority, whether during the prenatal period or in the three months following birth [21]. Program implementation fidelity, including addressing questions of affect and depression, seems to have taken second place with regard to more pressing issues around supporting mothers to resolve pressing social, financial or practical problems during the perinatal period. This remained true despite the fact that mothers with higher EPDS scores at three months postpartum tended to have received a greater number of home visits than less depressed mothers.

A third limitation was that, although all stakeholders, were blinded to assignment at inclusion, it is likely that mothers in the intervention arm would have mentioned to outcome assessors the fact that they were receiving home visits, thus potentially influencing outcome scores. This phenomenon, common to many community-based projects comparing a specific intervention with regard to usual care, may introduce a bias that needs to be taken into account in interpreting the final results.

Finally, it must not be forgotten that, although the intervention was implemented to reduce postnatal depression, for example using specifically trained psychologists with weekly supervision to discuss difficult situations, including handling mothers with depression, the current results are based on EPDS screening for self-declared depressive symptoms and not on formal diagnoses of depression, performed by qualified psychiatrists. In spite of the fact that the EPDS has been validated in local French language samples for both prenatal and postnatal depression, reported prevalence rates in the present study can only be considered to be estimates.

## Conclusion

The present study evaluated the impact on PND symptomatology of a multifocal perinatal home-visiting intervention using trained, supervised psychologists and targeting the major modifiable determinants of infant mental health, including PND, in a sample of women presenting risk factors associated with subsequent mental health problems in their children. Similarly to many other multifocal interventions targeting high-risk populations, CAPEDP failed to demonstrate an overall impact on PND symptomatology. However, it did appear to be effective in terms of primary prevention of PND and for reducing PND levels among subgroups of women with a higher educational level or who were planning to raise their child with the father. Effective overall reduction of PND for young, first-time mothers presenting multiple psychosocial risk factors may require more tailored interventions addressing the specific problems of women such as those planning to bring up their child alone, those with a lower educational level or those who already present depressive symptoms during pregnancy. Furthermore, the high program drop-out rate, significantly associated with women presenting multiple risk factors for their future child’s mental health, indicates that further research is needed to develop programs that are both adapted to and attractive to this important subgroup. This may well have significant implications for current home-visiting policy within the health and social perinatal care system in France, particularly in terms of adapting interventions to the needs of specific populations.

## Supporting Information

Checklist S1 **CONSORT Checklist.**
(DOC)

Protocol S1 **Trial Protocol.**
(DOC)
