# Supplementary material for: Impact of a Manualized Multifocal Perinatal Home-Visiting Program Using Psychologists on Postnatal Depression: The CAPEDP Randomized Controlled Trial
Source: PLoS One. 2013 Aug 19;8(8):e72216. doi: 10.1371/journal.pone.0072216 (PMC3747116; doi:10.1371/journal.pone.0072216)
Supplement: Protocol S1 — Trial Protocol. (DOC) [file pone.0072216.s002.doc]

Compétences parentales et Attachement dans la Petite Enfance - Diminution des risques liés aux troubles de santé mentale et Promotion de la résilience

Projet CAPEDP

Pr. GUEDENEY – Service de Psychopathologie de l’Enfant et de l’Adolescent

BICHAT – Claude Bernard

Tel : 01 42 55 03 09

Fax : 01 42 52 29 72

Mail : antoine.guedeney@bch.ap-hop-paris.fr


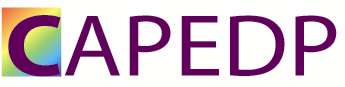


P051045 – AOM 05056

***Département de la Recherche clinique et du Développement - Hôpital Saint-Louis - 1 av. Claude Vellefaux 75010 Paris***

***Version mai 2006***

**Equipes associées au projet**

- Pr Antoine Guédeney, Dr Romain Dugravier du Groupe Hospitalier Bichat-Claude Bernard, service de psychopathologie de l'enfant et de l'adolescent, intersecteur de psychiatrie infanto-juvénile du 18^e^ arrondissement de Paris ;
- Dr Florence Tubach du Groupe Hospitalier Bichat - Claude Bernard, département d'Epidémiologie, et de Recherche Clinique ;
- M. Tim Greacen, M. Thomas Saïas du Laboratoire de recherche de l’Etablissement public de santé Maison Blanche ;
- Dr Alain Haddad, chef de service de l’intersecteur de psychiatrie infanto-juvénile du 19^e^ arrondissement de Paris, Etablissement public de santé Maison Blanche ;
- Dr Bertrand Welniarz chef de service d’intersecteur de psychiatrie infanto-juvénile de l’Etablissement public de santé Ville-Evrard ;
- Dr Diane Purper-Ouakil, pédopsychiatre à l’hôpital Robert-Debré, service du Pr Mouren-Simeoni ;
- Pr Dominique Mahieu-Caputo du Groupe Hospitalier Bichat - Claude Bernard, service de gynécologie obstétrique du Pr P Madelenat ;
- Dr Bernard Topuz, Dr Lucile Rivera des Services de Protection maternelle et infantile (PMI) de la Seine-Saint-Denis ;
- Pr Richard E. Tremblay, Pr Sylvana Côté, Nathalie Fontaine de l’Université de Montréal ;
- Pr George M. Tarabulsy, Pr Michel Boivin, de l’Université Laval, à Québec.

##### Unité de Recherche Clinique

Dr Florence Tubach

Département d'Epidémiologie, Biostatistique et Recherche Clinique
Groupe hospitalier Bichat-Claude Bernard
46 rue Henri Huchard
75018 Paris

Tél : 01 40 25 62 54

Fax : 01 40 25 67 73

#### Promoteur

AP-HP représentée par la DRCD

Délégation Régionale à la Recherche Clinique

Hôpital Saint-Louis

1 avenue Claude Vellefaux, 75010 Paris

Tél : 01 44 84 17 65

Fax : 01 44 84 17 99

TABLE DES MATIERES

[*Résumé du protocole 4*](#__RefHeading___Toc137005799)

[1. Etat de la question et objectif de la recherche 5](#__RefHeading___Toc137005800)

[Introduction 5](#__RefHeading___Toc137005801)

[Les conditions de l’efficacité de l’intervention : modèles théoriques 7](#__RefHeading___Toc137005802)

[Le contexte français 8](#__RefHeading___Toc137005803)

[2. Objectifs de la recherche 9](#__RefHeading___Toc137005804)

[Hypothèses testées 9](#__RefHeading___Toc137005805)

[Objectifs primaires 9](#__RefHeading___Toc137005806)

[Objectifs secondaires 9](#__RefHeading___Toc137005807)

[3. Plan Expérimental 10](#__RefHeading___Toc137005808)

[Choix du plan expérimental et justification 10](#__RefHeading___Toc137005809)

[Randomisation 10](#__RefHeading___Toc137005810)

[4. Critères de sélection des personnes 10](#__RefHeading___Toc137005811)

[Critères d’inclusion 10](#__RefHeading___Toc137005812)

[Critères de non inclusion 10](#__RefHeading___Toc137005813)

[Mode de recrutement 11](#__RefHeading___Toc137005814)

[Durée de participation de chaque personne incluse dans la recherche 11](#__RefHeading___Toc137005815)

[5. Intervention évaluée 11](#__RefHeading___Toc137005816)

[6. Critères d’évaluation 13](#__RefHeading___Toc137005817)

[Critères de jugement principaux 13](#__RefHeading___Toc137005818)

[Critères de jugements secondaires 13](#__RefHeading___Toc137005819)

[Variables mesurées 14](#__RefHeading___Toc137005820)

[7. Schéma et conduite de la recherche 17](#__RefHeading___Toc137005821)

[Signature du consentement 17](#__RefHeading___Toc137005822)

[Suivi des sujets 18](#__RefHeading___Toc137005823)

[Supervision des intervenants et des évaluateurs 18](#__RefHeading___Toc137005824)

[L’attitude à avoir en cas de situation d’urgence et le monitoring attenant 18](#__RefHeading___Toc137005825)

[Comité scientifique 18](#__RefHeading___Toc137005826)

[Durée totale prévisionnelle de la recherche 18](#__RefHeading___Toc137005827)

[Règles d’arrêt 18](#__RefHeading___Toc137005828)

[8. Nombre de personnes et justification 19](#__RefHeading___Toc137005829)

[9. Analyse statistique 19](#__RefHeading___Toc137005830)

[Gestion des données et analyse statistique 19](#__RefHeading___Toc137005831)

[10. Aspects logistiques légaux et généraux 20](#__RefHeading___Toc137005832)

[11. Retombées attendues scientifiques et en termes de santé publique 24](#__RefHeading___Toc137005833)

[BIBLIOGRAPHIE 25](#__RefHeading___Toc137005834)

[Annexe 1a : Formulaire d’information et de consentement 29](#__RefHeading___Toc137005835)

[Annexe 1b : Formulaire d’information et de consentement 2 31](#__RefHeading___Toc137005836)

[Annexe 1c : Formulaire d’information et de consentement 3 34](#__RefHeading___Toc137005837)

[Annexe 1d : Formulaire d’information et de consentement 4 36](#__RefHeading___Toc137005838)

[Annexe 2 : Carte provisoire de la zone d’étude 39](#__RefHeading___Toc137005839)

[Annexe 3 : Chronologie des visites 40](#__RefHeading___Toc137005840)

[Annexe 4 : Liste des centres participants à l’étude et des investigateurs. 41](#__RefHeading___Toc137005841)

# Résumé du protocole

Les études épidémiologiques soulignent la fréquence des troubles de santé mentale dans la petite enfance. Les troubles du comportement ou le retrait peuvent apparaître de manière importante dès la deuxième année des enfants, témoignant de leur souffrance psychique. Lorsque ceux-ci évoluent dans un contexte psychosocial vulnérable, ces comportements risquent de se pérenniser. Le projet actuel consiste à évaluer, dans un contexte urbain français, l’impact d’une intervention centrée sur la prévention des risques psychosociaux et la promotion de la résilience en ce qui concerne la santé mentale de la petite enfance.

**Objectifs :**

Evaluer l’impact d’un programme de prévention des troubles psychologiques et de promotion de la santé mentale de la petite enfance en termes de :

- dépression post-natale,
- compétences parentales,
- troubles psychologiques de l’enfant : les troubles externalisés, repérables à 24 mois, et le retrait relationnel, repérable à 18 mois.

Ces critères de jugement principaux sont des éléments prédictifs majeurs de l’efficacité de l’intervention à plus long terme chez l’enfant et l’adolescent^93^.

**Plan expérimental** : essai d’intervention randomisé en deux bras parallèles, ouvert et utilisant la méthodologie PROBE (*Prospective Randomized Open Blinded Endpoint study).* Cet essai comparera l’intervention aux soins usuels.

**Nombre de sujets nécessaire** :  880 (440 mères + 440 enfants)

**Critères d’éligibilité** : femmes primipares, âgées de moins de 26 ans et ayant ou un faible niveau d’éducation, ou se déclareront isolées durant leur grossesse ou bénéficieront de la CMU.

**Intervention évaluée :** Il s’agit d’une intervention centrée sur la prévention des risques psychosociaux et la promotion de la résilience en ce qui concerne la santé mentale de la petite enfance. Elle consiste en des visites à domicile débutant avant la 27^e^ semaine de grossesse et se poursuivant jusqu’aux deux ans de l’enfant. Exercées par des psychologues formés*,* ces visites auront pour but de renforcer les compétences parentales, d’optimiser les liens avec les services sanitaires et sociaux, de favoriser la qualité des relations entre l’enfant et sa mère et de réduire les facteurs de risque modifiables.

**Schéma de la recherche :** Des visites d’évaluation à l'inclusion, ensuite lorsque l'enfant aura 3, 6, 12, 18 et 24 mois seront réalisées par des évaluateurs en insu du bras de randomisation.

**Durée de participation pour chaque personne ayant accepté de participer à la recherche :** 2 ans et 8 mois pour les mères, 2 ans et 2 mois pour les enfants

**Durée totale de la recherche** : 50 mois

**Critères de jugements principaux :**

- La survenue d’une dépression postnatale : le diagnostic sera défini par un score de l’*Edinburgh Post-partum Depression Scale* (EPDS) supérieur à 11.

- L’acquisition des compétences parentales : le critère de jugement sera l’échelle *Home Observation for the Measurement of the Environment* (HOME).

- La prévalence des troubles psychosociaux : le critère de jugement sera la *Child Behavior Checklist* (CBCL).

**Analyse statistique principale**

L’analyse principale se fera en intention de traiter.

- Le pourcentage de femmes ayant une dépression postnatale sera comparé entre les deux groupes par un test de Chi2.
- Compétences parentales : on comparera le score moyen HOME mesuré à 24 mois entre les 2 bras par un test de Student.
- Troubles de santé mentale et du comportement : on comparera le score moyen CBCL mesuré chez les enfants à 24 mois entre les 2 bras par un test de Student.

# 1. Etat de la question et objectif de la recherche

## Introduction

L’ensemble des théories développementales reconnaissent que l’environnement familial et les relations parents-enfants^1,2,3^ ont des effets à long terme sur le fonctionnement psychologique des individus^4,5^. Le développement d’un attachement précoce sécure, permettant à l’enfant d’explorer son environnement en sécurité, contribuerait à la mise en place d’un large éventail de compétences, dont le goût pour l’apprentissage, l’estime de soi, des compétences sociales affirmées, des relations interpersonnelles durables, voire de solides compétences cognitives^6^. Ces enfants développeraient ainsi une capacité dite de *résilience* face aux événements de vie stressants. Les enfants ayant grandi dans des familles sécurisantes et émotionnellement équilibrées apprennent plus facilement à gérer leurs émotions et à faire face efficacement aux problèmes quotidiens^7^. De même, les nourrissons sont particulièrement sensibles aux contextes familiaux difficiles. Les troubles de santé mentale des parents (notamment la dépression), la violence intra-familiale, les contextes familiaux à vulnérabilités multiples, sans relations stables, peuvent amener les jeunes enfants à se développer selon des modalités révélatrices de leur souffrance^6, 8^.

Les troubles de santé mentale de la petite enfance peuvent se manifester précocement par des comportements problématiques, tels que le repli sur soi, l’inhibition, mais également -et davantage- l’agitation ou l’agressivité. Ces comportements peuvent se pérenniser si les difficultés de l’enfant et/ou de son environnement ne sont pas prises en compte de façon précoce.

Ainsi, suite à un récent rapport du ministère de la santé sur la violence chez les jeunes, notamment parmi les enfants les plus jeunes^9^, une enquête du Sénat^10^ recommande la mise en place dans les maternités d’unités d’accompagnement psychologique lors de la grossesse et de l’accouchement, l’exploration de pistes pour la recherche, pour l’action et pour la recherche-action, le renforcement des moyens de la Protection Maternelle et Infantile (PMI) et la mise en place d’actions systématisées de promotion de la santé mentale. Effectuant ce même constat au niveau international, l’Organisation mondiale de la santé (OMS) recommande vivement la mise en place et l’évaluation d’actions de prévention et d’intervention précoce^11^. En effet, plusieurs études internationales longitudinales - au Québec^12^ et aux États-Unis^13, 14^ - ont montré que l’agressivité physique peut se manifester très tôt, avec un pic de comportements violents entre 17 et 24 mois ; plus tard, par le biais des demandes éducatives, de l’acquisition de nouvelles compétences sociales, les enfants ont moins recours à la violence physique^12^. Toutefois, dans une proportion faible mais non négligeable, certains présentent des comportements agressifs persistants^15-23^. Des études récentes ont démontré que les enfants avec des scores élevés pour les troubles externalisés sur la *Child Behavior Checklist* (*CBCL 1½-5*) à 24 mois seraient plus à risque de présenter un diagnostic de troubles des conduites ou oppositionnel à 5 ans^24^. Si ce phénomène ne peut évidemment être extrapolé pour la suite de leur développement, tous les enfants présentant des troubles du comportement ne devenant pas délinquants, de nombreuses études montrent néanmoins que les adolescents et jeunes adultes violents étaient souvent des enfants très agressifs^25-27^, ce phénomène étant particulièrement ressenti en milieu urbain^13, 28-30^.

##### Les facteurs de vulnérabilité

Une littérature internationale importante montre que certains facteurs ou groupes de facteurs de vulnérabilité précoce (observables dans les premiers mois de vie, voire avant la naissance) sont associés aux contextes de pérennisation de ces comportements violents^28, 31^. Ces facteurs sont liés :

- au **quartier** : le nombre de mois passés dans un quartier caractérisé par la « désorganisation communautaire » avec taux de criminalité élevé, présence de drogues ou d’armes, logements de piètre qualité ^28, 32-34^ ;
- au **contexte familial** : manque de ressources financières (mais uniquement en présence d'autres facteurs de risque^35^), manque d’interaction entre les parents^14, 28^, utilisation de techniques éducatives coercitives ^14, 19, 36^, famille monoparentale ^14, 37^, histoire familiale d’abus et de violence ^28^, attitude hostile de la mère, particulièrement vis-à-vis des garçons ^38^, mobilité résidentielle ^39^, attitude parentale peu sensible ^40^ ;
- à **l’histoire personnelle de la mère ou du père** : jeunesse de la mère ^14, 22, 27^, manque d’expérience maternelle (mères primipares) ^14, 22^, tabagisme maternel ^14, 22, 41^, histoire de comportement antisocial chez la mère à l’école ^12, 27^, troubles de conduite chez un parent ^20^, mère ayant un bas niveau d'éducation ^42^, parents et surtout la mère présentant une personnalité antisociale ^12, 42, 43^, parents ayant une histoire de criminalité ^39^, parents présentant des troubles liés à l'alcool ou à la drogue ^39^ ;
- à la **dépression maternelle pré et postnatale** ^44^. La dépression maternelle du post-partum (touchant 13-15% des femmes avec un premier pic de deux à trois mois après l’accouchement^45^, et un autre 6 mois après l’accouchement^46^) serait en cause dans l’apparition de certains troubles de l’attachement et jouerait un rôle particulier dans les troubles du comportement,,, à travers des interactions mère-enfant dysharmoniques, caractérisées pour l’enfant par le retrait, l’hostilité ou l’impulsivité^44, 47-51^. La dépression maternelle a fait l’objet de nombreuses interventions de prévention précoce^52^. Les évaluations soulignent l'intérêt d’entamer l'intervention durant la grossesse de façon à ce qu’elle soit en place avant la période critique du post-partum, les mères déprimées refusant dans une large proportion d’être prises en charge par un professionnel inconnu. D’autre part, l’intervention pendant la grossesse a une valeur préventive quant à la survenue d’une dépression du post-partum^53, 54^ et, si malgré cela une dépression survient, l’intervention préalable a une valeur thérapeutique démontrée ^54^ ;
- à **l’histoire de l’enfant** : abus et négligence ^55, 56^, présence d’autres troubles de santé mentale chez l'enfant ^28, 57^, troubles ou retards de développement du langage ^58^ ;
- aux **troubles précoces de l’attachement**: L’attachement[[1]](#footnote-2) dit insécure, et particulièrement l’attachement désorganisé, est un facteur de vulnérabilité quant à la survenue de troubles du comportement^59, 60^ ;
- à **des facteurs génétiques** : Si le rôle des facteurs génétiques dans les capacités de régulation émotionnelle et de gestion de l’agressivité est maintenant bien reconnu, le phénotype impliqué dans les troubles agressifs reste peu déterminé, comportant des aspects liés d'une part aux problèmes de maîtrise de compétences (comportement pro-social, capacité de contrôler son comportement oppositionnel ou agressif, développement du langage oral) et d'autre part à la présence d'autres troubles caractérisés (hyperactivité avec troubles d’attention, trouble des conduites) ^58, 61-65^.

**Une référence : l’étude de David Olds à Elmira**

Constatant l’importance du contexte social et de l’environnement familial pour l’enfant, les professionnels de la petite enfance de toutes les disciplines ont commencé à s’intéresser aux facteurs contribuant à l’amélioration des relations de l’enfant avec sa famille.

L'étude Elmira (menée par David Olds et son équipe), pionnière en matière de prévention des troubles du comportement de la petite enfance, s’adressait à des femmes enceintes présentant des vulnérabilités multiples : jeunes, primipares, célibataires et ayant peu de ressources financières^22, 67^ .

L’intervention, initiée de façon très précoce (avant le troisième trimestre de la grossesse) consistait en des visites à domicile se prolongeant jusqu’au 24^e^ mois ; l’objectif étant d’intervenir sur les facteurs de risque précoces potentiellement modifiables (consommation d’alcool, de tabac…) et de favoriser l’émergence de facteurs de protection (tels que la promotion des compétences parentales).

Il a été montré que la réussite de l’intervention dépendait non seulement du contenu mais également de la fréquence des contacts entre la femme et l'intervenant, avec comme objectif principal la création d’une alliance thérapeutique, c’est-à-dire la capacité de la femme et de l'intervenant à élaborer conjointement une stratégie de prise en charge satisfaisante pour l’un comme pour l’autre. Les évaluations effectuées au cours de l’intervention ainsi qu’au 24^e^ mois mettent en évidence des effets positifs sur tout un ensemble des facteurs de vulnérabilité psychosociale ciblés :

le comportement et le devenir maternels : moindre consommation de tabac et de recours aux services d’urgence, moins de grossesses et de naissances ultérieures dans les 24 mois, meilleure utilisation des services de prévention, niveau d’études et d’insertion professionnelle plus élevé,

l’interaction mère-enfant et la qualité de vie au domicile,

le devenir de l’enfant ; à 6 mois : moindre vulnérabilité aux stimuli effrayants, vitalité accrue aux stimuli de joie et de colère, tempérament moins irritable selon la mère ; à 21 mois : moindre taux de retard de langage ; à 24 mois : moindre prévalence de retard de développement mental et de problèmes de comportement^68^.

De plus, la modification constatée des facteurs de vulnérabilité psychosociale précoces par rapport au groupe contrôle est associée à des effets bénéfiques ultérieurs, d’abord en termes de troubles du comportement chez l’enfant, mais également de comportements antisociaux ultérieurs (moins d’arrestations, de condamnations, de fugues, de consommations d’alcool et de tabac pendant l’adolescence)^69^. Les évaluations mettent également en évidence des effets positifs pour la mère : par rapport aux mères ayant bénéficié des suivis médico-sociaux usuels, les mères participant au programme expérimental présentaient des bénéfices en termes de santé mentale, un moindre recours aux aides sociales, une prévalence moindre de comportements antisociaux, de maltraitance et de négligence de l’enfant, une moindre consommation d’alcool et de drogues.

La question de la reproductibilité de ce type d’études dans d’autres contextes a été posée. En effet, dans de nombreux pays développés, les familles bénéficient de systèmes de soutien sanitaire, social et éducatif plus généralisés que ceux proposés aux Etats-Unis. En France, l’efficacité des interventions généralistes, notamment de type PMI, en termes de prévention des troubles de la santé mentale n’a jamais été évaluée de façon contrôlée.

##### La réplication de l’étude d’Elmira dans d’autres contextes sociaux et nationaux

Le programme Elmira, qui s’était déroulé dans un contexte semi-rural, a été répliqué à Memphis^70^ et à Denver^71^, confirmant ainsi son efficacité en milieu urbain nord-américain auprès de différentes populations. En France, il n'existe pas d'intervention à large échelle ciblant les troubles de santé mentale de la petite enfance de façon spécifique. L’implantation de ce type de programme dans des contextes nationaux où les soins usuels, y compris chez les familles en situation de précarité, sont plus généralisés et accessibles que sur les sites américains, a néanmoins prouvé l’efficacité de cette approche^72, 73^. Ainsi, Davis et coll.^74^ dans une étude similaire basée sur des visites à domicile sur cinq sites européens découvrent au 24^e^ mois une meilleure qualité de vie familiale à domicile (mesurée par *l’Inventaire* *HOME*), un niveau moindre de stress dans l’interaction parent-enfant (*Index du stress parental*), une meilleure qualité générale de la relation parent-enfant, davantage de plaisir partagé dans l’interaction parent-enfant évaluée sur enregistrement vidéo et moins de problèmes de dépendance chez l’enfant.

## Les conditions de l’efficacité de l’intervention : modèles théoriques

**La psychologie communautaire : la notion d’empowerment**

**L'*empowerment* est une notion caractérisant la façon par laquelle l'individu accroît ses habiletés favorisant l'estime de soi, la confiance en soi, l'initiative et le contrôle^75^. Les notions de sentiment de compétence personnelle^76^, de prise de conscience^77^ et de motivation à l'action sociale^78^ y sont associées. L'*empowerment* comprend une dimension transactionnelle qui se joue aussi au plan social et collectif. Katz^79^, par exemple, le représente comme un paradigme synergique où les personnes sont interreliées, où il y a un partage des ressources et où la collaboration est encouragée.** Pour Rappaport^78^, l'*empowerment,* un objectif qui doit être au cœur de toute action de promotion de la santé, comporte quatre composantes : la participation, la compétence, l'estime de soi et la conscience critique individuelle et collective. Lorsque ces quatre composantes sont en interaction, un processus d'*empowerment* serait alors enclenché. L’action de promotion de la santé se centre sur les forces, les droits et les compétences des individus et de la collectivité, plutôt que sur les déficits ou les besoins^80^. Ainsi, dans une perspective d'*empowerment,* les intervenants en ville doivent garder à l'esprit que le système de soutien social et sanitaire à privilégier est celui dans lequel le sujet peut exercer un contrôle direct sur les décisions et les événements de sa vie quotidienne^78^. Il s'agit de reconnaître l'expérience subjective des personnes comme expertes de leur propre vie^81^ et de s’y appuyer.

**Le modèle écologique**

L’intervention de promotion de la santé qui cible l’*empowerment* du sujet ne peut être efficace sans prendre en compte les multiples strates de son contexte de vie. Le modèle écologique d’intervention, développé par Bronfenbrenner (1979)^2^, propose une vision de l’individu en interaction avec un environnement complexe divisé en strates proximales et distales. Au point de vue développemental, l’enfant est influencé par la manière dont ses parents l’élèvent (strate relationnelle proximale), par le milieu dans lequel il est élevé (strate relationnelle distale), mais également par ses caractéristiques propres (strate individuelle). Ces strates interagissent également : la manière dont un parent va prendre soin de son enfant va être déterminée par les relations entretenues par ce parent avec son milieu social (et avec les stress qui le caractérisent). A partir de cette théorie, les intervenants à domicile des projets de prévention précoce^82^ ont pour objectif de favoriser l’environnement social et relationnel de la famille, en impliquant un grand nombre de personnes dans le développement de l’enfant, mais aussi en favorisant l’accès aux services médico-sociaux pour l’ensemble de la famille^82^.

**La psychologie développementale et la théorie de l’attachement**

Si la prise en compte du contexte écologique du sujet est déterminante pour l’efficacité de l’intervention, la manière dont le sujet apprend à nouer des liens psychosociaux, et notamment la notion d’*attachement*, doit faire l’objet d’une attention particulière. Pour Bowlby^83^, l’attachement est un lien affectif durable d’un enfant envers un adulte qui en prend soin. L’attachement du bébé à la mère (ou son substitut) lui offre un sentiment de sécurité ainsi qu’une capacité à explorer le monde. La qualité de ces premiers liens forme un modèle interne opérant, c'est-à-dire un modèle de compréhension des relations sociales. Ce modèle constituera jusqu’à l’âge adulte une manière d’appréhender les relations sociales (en quantité et qualité).

Les théoriciens de l'attachement proposent un modèle aménagé sur des représentations intériorisées se fondant sur l'expérience passée avec ses figures d'attachement^4, 83-85^. Prises ensemble, ces représentations peuvent être conçues comme une idée représentative ou un modèle cognitif de la relation. Aspect de l'image de soi, espérance de comportements de la part des autres et impression émotionnelle implicite de la relation sont autant d'éléments qui font partie de ce modèle^86^. Ce modèle est initialement développé lors des premières interactions mère-enfant et évolue au fil de nouvelles expériences avec ses figures d'attachement. Cependant, il reste dépendant du premier *modèle de représentations* de ce qu'est une relation et des premières structures cognitives s'y rattachant^87^. Les travaux de Mary Ainsworth ont défriché le domaine des relations d'attachement et ont donné lieu aux catégories d'attachement généralement reconnues. Son modèle expérimental, la *Situation Etrange* lui a permis d'observer et de hiérarchiser divers schèmes de comportement^88^. Grâce à ce mode d'observation, Ainsworth a étudié le niveau d'équilibre entre les comportements d'attachement et d'exploration adoptés par des tout-petits (12 à 20 mois) soumis à un stress modéré. Elle en a déduit trois formes principales d’attachement (corrélées de façon significative à la sensibilité maternelle) :

- l’attachement de type sécure s’accompagne, chez l’enfant, d’une meilleure estime de soi et de la capacité de faire appel lorsqu’il en a besoin. Il favorise également la capacité d’exploration. L’enfant manifeste une forme de protestation lors des séparations et accueille sa mère avec plaisir, à son retour.
- l’attachement de type insécure évitant : l’enfant ne fait pas appel à autrui au fur et à mesure que son stress augmente. Il a tendance à masquer sa détresse émotionnelle, ou à se sentir invulnérable, et à considérer que l’on ne peut pas faire confiance aux autres. Il essaie de garder le contrôle dans les situations de détresse en diminuant la réactivité du système d’attachement et en réduisant ses signaux de détresse en direction des parents.
- l’attachement de type insécure ambivalent ou résistant. L’enfant se montre très ambivalent en situation de stress, comme s’il résistait à son besoin d’être réconforté. Il adopte une stratégie d’augmentation de fonctionnement du système d’attachement et d’augmentation des signaux. Il manifeste de la détresse lors de la séparation, un mélange de recherche de contact et de rejet coléreux, des difficultés à être réconforté.

Les études longitudinales américaines et allemandes^89, 90^ ultérieures sur l’attachement ont montré la capacité prédictive de l’attachement dit « sécure », en terme de relation avec les pairs, d’aisance sociale, de stabilité de l’attention, d’affect positif, de curiosité, de capacité d’exploration, de capacité de résilience et d’empathie. Par ailleurs, l’attachement sécure n’est pas fixé la vie durant : il peut devenir insécure si les conditions d’environnement changent (traumatismes, deuils…), et inversement. Précisons que les catégories insécures, qu’elles soient résistantes ou évitantes, correspondent à des stratégies adaptatives. Elles ne sont pas, en elles-mêmes, synonymes de pathologie.

Plus récemment, George, Main et Kaplan^91^ ont élaboré un nouvel outil d’évaluation l*’Adult Attachment Interview.* C’est un entretien semi-structuré destiné aux adultes qui porte sur l’état d’esprit actuel de la personne interrogée vis-à-vis de l’attachement. L’analyse du discours plus que le contenu permet de classer les récits sur les expériences d’attachement. Les auteurs font correspondre des figures parentales aux différentes catégories d’attachement de l’enfant identifiées avec la *Situation Etrange*. Ainsi, aux enfants classés comme *insécures évitants* correspondent des figures parentales détachées vis-à-vis de leurs propres expériences d’attachement ; aux enfants *insécures ambivalents* correspondent des figures parentales préoccupées ; aux enfants *sécures* correspondent des figures parentales libres et autonomes. De là, les auteurs introduisent une autre catégorie, l’attachement *désorganisé*, (enfants avec une stratégie incohérente) correspondant chez les adultes à la catégorie « *non résolue*» en lien avec un deuil ou un traumatisme. La désorganisation correspondrait à un conflit entre deux stratégies incompatibles et se traduirait par une interruption prématurée du comportement d’attachement ou par l’activation simultanée de comportements contradictoires de recherche et de fuite, ou encore par des manifestations d’effroi. Cette incapacité à développer une stratégie comportementale organisée serait due à l’impossibilité pour l’enfant de trouver une protection auprès de sa figure d’attachement. En effet, ces enfants ont en général subi davantage de violences ou d’abus physiques ou sexuels ou ont davantage été exposés de façon terrifiante à des parents eux-mêmes terrifiés que les enfants présentant des stratégies attachementales. L’enfant est alors dans une situation paradoxale, puisqu’il est pris entre son besoin d’attachement et de sécurité, et la peur vis-à-vis d’un parent maltraitant ou lui-même désorganisé. Ainsi, pour Shaw et Vondra^59^, à partir d’un échantillon de 100 jeunes enfants ayant de multiples facteurs risque, la qualité de l’attachement à 12 mois était un facteur de prédiction des problèmes de comportements à l’âge de 3 et 5 ans : 60 % des enfants classés comme désorganisés à l’âge de 12 mois montraient des niveaux cliniques d’agression, alors que 31 % seulement des évitants, 28 % des ambivalents et seulement 17 % des enfants sécures montraient de tels niveaux^92^.

## Le contexte français

Le système actuel de prévention repose sur la Protection maternelle et infantile (PMI), une offre de soins préventifs sur l'ensemble du territoire proposant un suivi généraliste par une équipe pluridisciplinaire dès la naissance, voire avant dans certains cas. En ce qui concerne la santé mentale, les stratégies restent a priori généralistes, avec un objectif de soutien à la parentalité et à la relation mère-enfant et de signalement et d’orientation de cas problématiques.

Pour prendre l’exemple de la Ville de Paris, plus de 20% des enfants nés chaque année et signalés par la maternité comme présentant un ou plusieurs facteurs de risque somatiques ou psychosociaux sont adressés dès les premiers jours de vie aux équipes de la PMI. Si, en 2002, la majorité des mères (54%) n’était vue qu’une seule fois à domicile, seulement 7% l’étaient plus de trois fois, de nombreuses familles demeurant utilisatrices des services d’accueil et de consultation en ambulatoire proposés dans les centres PMI de proximité.

Quoi qu’il en soit, certains auteurs affirment que le système en son entier n’a jamais été véritablement évalué ; pour Ikounga Ngoma et Brodin (2001), par exemple, il est clair que nous savons peu sur son efficacité quant à la réduction des risques de troubles psychosociaux de l’enfant et de troubles relationnels précoces, bien que le développement de ces études soit encouragé par la politique française de santé mentale en faveur des enfants et adolescents (Circulaire n°70 du 11 décembre 1992).

# 2. Objectifs de la recherche

Ce projet de recherche a pour but d’évaluer l’impact dans un contexte urbain français d’une intervention visant à la réduction des risques de troubles relationnels précoces et de troubles psychosociaux de la petite enfance et à promouvoir les facteurs de résilience (insertion sociale, alliance thérapeutique, sécurité de l’attachement). 440 mères seront recrutées en maternité et randomisées en deux bras. Le premier bénéficiera des soins communautaires habituellement dispensés et rencontrera un évaluateur à 6 reprises. Le second bras bénéficiera de visites à domicile, mises en place dans l’objectif de renforcer les compétences parentales et le sentiment de compétence parentale, d’optimiser leur lien avec les services sanitaires et sociaux (PMI, crèches, bureaux d’aide sociale…) et de réduire les facteurs de vulnérabilité psychosociale modifiables. Ce suivi à domicile continuera jusqu’au 24^e^ mois de l’enfant. Les sujets de ce second bras seront également rencontrés à 6 reprises par des évaluateurs indépendants. L’étude proposée s’inscrit dans le cadre d’un programme de recherche plus ample qui évaluera, entre autres, l’impact de l’intervention jusqu’à la fin de l’adolescence pour mettre en évidence les effets préventifs à long terme sur la santé mentale.

## Hypothèses testées

- Un programme de prévention des troubles psychologiques et de promotion de la santé mentale impliquant des visites à domicile systématiques dès le deuxième semestre de la grossesse et jusqu’au 24^e^ mois de l’enfant par des intervenants formés peut être mis en place de façon effective dans un contexte urbain français.
- Cette intervention ciblée :
  - favorisera les compétences parentales ;
  - diminuera l’incidence de la dépression post-natale ;
  - diminuera l’incidence de troubles de santé mentale.

## Objectifs primaires

L’objectif de cette étude est donc d’évaluer l’impact d’un programme de prévention des troubles psychologiques et de promotion de la santé mentale en termes de :

- dépression post-natale,
- compétences parentales,
- troubles psychologiques de l’enfant : les troubles externalisés, repérables à 24 mois, et le retrait relationnel, repérable à 18 mois.

Ces critères de jugement principaux sont des éléments prédictifs majeurs de l’efficacité de l’intervention à plus long terme chez l’enfant et l’adolescent^93^.

## Objectifs secondaires

Evaluer l’impact de cette intervention ciblée de prévention par rapport aux soins usuels, sur :

- la qualité de l’attachement de l’enfant,
- la compréhension et l’utilisation adéquate du système sanitaire, éducatif et social,
- le réseau social et le soutien social perçu par les mères,
- le sentiment de compétence parentale,
- le développement psychomoteur de l’enfant,
- les connaissances sur le développement de l’enfant,
- le stress parental,
- l’inscription sociale de la mère en termes d’activité éducative et professionnelle.

L’adhésion au programme de prévention ainsi que la création d’une alliance thérapeutique de qualité seront aussi évaluées pour le groupe bénéficiant de l’intervention.

# 3. Plan Expérimental

## Choix du plan expérimental et justification

Il s’agit d’un essai d’intervention randomisé en deux bras parallèles, ouvert et utilisant la méthodologie PROBE (*Prospective Randomized Open Blinded Endpoint study* : ni le sujet ni l’intervenant ne sont en aveugle du bras de randomisation, mais celui qui recueille le critère de jugement est en aveugle).

Les essais randomisés restent à ce jour la méthode la plus rigoureuse pour déterminer une relation de cause à effet entre une intervention et les résultats observés chez les sujets. Eux seuls peuvent fournir une évaluation non biaisée de l’effet de cette intervention, la randomisation ayant pour effet de supprimer toute influence du sujet ou du médecin sur l’attribution de l’intervention et donc de ne pas orienter la sélection des sujets en fonction de l’intervention qu’ils vont recevoir.

Cette étude comparera :

- - un groupe de femmes primipares bénéficiant des soins usuels : prévention généraliste assurée par la PMI, services de soins (médecine générale, pédiatrie…) et systèmes d’aide sociale.
  - un groupe de femmes primipares bénéficiant en sus d’une stratégie de prévention précoce par le biais de visites à domicile pendant le troisième trimestre de grossesse et les 2 premières années de vie de l’enfant : visites à domicile à rythme intensif au départ, et réalisées par des intervenants formés sur la promotion de la résilience et la réduction de risques associés aux troubles du comportement de la petite enfance. Les intervenants seront encadrés par des cliniciens assurant une supervision régulière.

## Randomisation

La randomisation sera centralisée par fax (Unité de Recherche clinique Paris Nord) ; la liste de randomisation par blocs sera générée aléatoirement par ordinateur.

# 4. Critères de sélection des personnes

## Critères d’inclusion

Au contraire des programmes « universels » de prévention et de promotion de la santé (concernant la totalité d’un groupe d’âge, et avec des objectifs généralistes), la réussite en termes de coût-efficacité d’un programme de prévention des troubles psychosociaux et de promotion de la santé mentale dépend du ciblage des populations vulnérables^94^.

Seront ainsi incluses consécutivement les femmes se présentant dans les maternités participant à l’étude, acceptant de participer à la recherche (voir annexe 1, formulaire de consentement) et remplissant les critères suivants :

- domiciliées dans la zone d’intervention (voir annexe 2),
- parlant suffisamment le français pour donner un consentement valide et bénéficier de l’intervention et répondre aux évaluations,
- jeunes (< 26 ans),
- primipares,
- moins de 27 semaines d’aménorrhée au moment de la première rencontre à domicile,
- affiliée à un régime de sécurité social ou bénéficiant de la Couverture Maladie Universelle,
- et…
  - ayant un niveau d'éducation inférieur au baccalauréat (ou à 12 années d’études pour les personnes n’ayant pas été scolarisées en France),

ET/OU

- - se déclarant isolées lors de leur entretien en maternité (défini par une question standardisée),

ET/ OU

- - bénéficiant de la Couverture Maladie Universelle.

## Critères de non inclusion

- femme dont le suivi est à priori impossible (gens du voyage non sédentarisés, réfugiés en transit, etc.),
- femme recevant un suivi soutenu pour une autre raison, telle qu’une pathologie nécessitant une prise en charge lourde : déficience ou maladie mentale, toxicomanie, maladie somatique avec suivi fréquent ; en effet, l’inscription médico-sociale et la création d’une alliance thérapeutique étant un des objectifs principaux de l’intervention, une inscription médico-sociale déjà existante pour une autre raison introduirait un biais important ; certaines de ces populations sont par ailleurs déjà ciblées dans la zone d’étude dans des programmes spécifiques (mères toxicomanes, mères présentant des troubles psychiatriques),
- personnes ne bénéficiant pas de la sécurité sociale (ou de la CMU) ; les personnes bénéficiant de l’Aide Médicale d’Etat ne pourront être incluses,
- refus de consentement.

## Mode de recrutement

- La plupart des femmes correspondant aux critères d'inclusion dans l'étude consultent dans les maternités participant à l’étude avant le 3è trimestre de grossesse, y compris les femmes en situation de précarité. Les maternités publiques sont donc le lieu idéal de recrutement.
- Un registre des patientes éligibles et non incluses sera tenu dans chaque centre.
- En raison de la durée importante de passation de l’outil *Situation Etrange* (à 12-15 mois) et du fait que, à la différence de l’ensemble des autres outils, il étudie directement le comportement de l’enfant, cet outil fera l’objet d’un protocole ancillaire particulier proposé à 50 femmes dans chaque bras de l’étude, avec une démarche de recherche de consentement spécifique (voir Annexe : formulaires de consentement spécifiques recueillant le consentement de la mère et du père pour les 100 familles en question et le consentement des parents des mères mineures).

## Durée de participation de chaque personne incluse dans la recherche

Chaque femme sera suivie à partir du second trimestre de grossesse au plus tard jusqu’aux 26 mois de l’enfant né de cette grossesse soit 32 mois .

# 5. Intervention évaluée

L’intervention est destinée à des femmes enceintes, jeunes, et avec un de trois critères : soit avoir un faible niveau d’instruction soit se déclarer isolées soit être dans une situation économique précaire. Elle s’ajoute à l’offre standard de services sanitaires, sociaux et éducatifs publics (services de PMI, de pédiatrie, aides sociales, crèches...). Elle sera adaptée au contexte français, standardisée pour pouvoir être ensuite répliquée et, le cas échéant, généralisée. Les modalités de l’intervention s’appuient sur le programme québécois du GRIP qui s’inspire de l’intervention *Nurse Family Partnership*^70, 98^ implanté aux Etats-Unis auprès de populations précarisées et ensuite dans certains pays européens. L’intervention consiste en des visites à domicile (VAD) régulières par des professionnels de la petite enfance.

*Fréquence des interventions à domicile*

Les VAD seront mises en place dès l'inclusion, et ce suivi à domicile continuera jusqu’au 24^e^ mois de l’enfant. L’effet de l’intervention étant proportionnel à l’intensité du suivi^40^, il s’agit d’inclure suffisamment de contacts pour créer une alliance thérapeutique avec les familles^70, 99^: les visites, hebdomadaires pendant la grossesse, diminuent progressivement jusqu’à environ une fois par mois dans la 2^e^ année[[2]](#footnote-3). La durée d'une visite à domicile est de 60 à 120 minutes. Suivant les protocoles des études internationales ayant montré une efficacité, il est prévu de réaliser :

- 3 visites[[3]](#footnote-4) durant le premier mois d’inclusion (7^e^ mois de grossesse),
- 1,5 visites par mois durant les deux derniers mois de grossesse,
- 2 visites pendant le 1^er^ mois de l’enfant
- 3 visites par mois durant les 2^ème^ et 3^ème^ mois de l’enfant,
- 2 visites par mois durant les trois mois suivants (jusqu’au 7^e^ mois de l’enfant),
- 1,5 visite par mois jusqu’aux 18 mois de l’enfant.
- 1 visite par mois jusqu’aux 2 ans de l’enfant.

*NB : La fréquence de ces visites pourrait être aménagée en fonction des besoins des familles.*

*Les intervenants*

- L’intervention s’appuie principalement sur des psychologues professionnels[[4]](#footnote-5), réputés plus efficaces que des bénévoles formés ^71^.
- La formation des intervenants joue un rôle déterminant^71^. Cette formation – dont une partie sera assurée par les partenaires québécois qui ont déjà mis en place un programme similaire en langue française - portera sur le contenu de l’intervention, les techniques de visite à domicile, la création et maintien d’une alliance thérapeutique, le réseau de soutien sanitaire et social. Chaque intervenant sera formé à l’ensemble du protocole d’intervention avant le début de celle-ci, mais également par le biais de formations continues.

*Contenu des visites à domicile*

Les interventions à domicile cibleront les facteurs psychosociaux et comportementaux qui pourront influencer le devenir de la mère et de l’enfant et se focaliseront sur les facteurs de vulnérabilité psychosociale effectivement modifiables en proposant des stratégies déjà éprouvées^31, 70, 102^.

L’intervention se structure en deux temps essentiels :

- un temps informel consacré à l’établissement d’un lien de confiance entre la participante et l’intervenant. Ce temps - généralement en début de séance - vise à assurer le développement d’un lien de proximité, qui ne peut se concevoir que dans l’élaboration d’une discussion informelle. A partir du modèle relationnel défini par Carl Rogers[[5]](#footnote-6), l’intervenant peut donc manifester ses capacités « de respect, d’authenticité, d’empathie, d’humilité, d’enthousiasme -modéré- et d’intégrité »^103^, facilitant le développement de ce lien, objectif principal de l’intervention, en ce qu’il conditionne la réussite de l’ensemble du processus.

🡺 On qualifiera ce temps de PROTOCOLE REACTIF

- un temps plus formel où l’intervenant peut aborder les différentes thématiques qui seront définies pour chacune des périodes de l’intervention (prénatal ; 0-3 mois ; 3-6 mois ; 6-12 mois ; 12-24 mois), et correspondant aux objectifs de recherche :

| **Réduire**  **-** | l’incidence de la dépression périnatale |
| --- | --- |
|  | l’incidence des troubles de santé mentale et notamment des troubles externalisés à 2 ans |
| **Favoriser**  **+** | les compétences parentales et le sentiment de compétence parentale |
|  | la sécurité de l’attachement |
|  | l’utilisation d’un réseau social personnel et le soutien social perçu |
|  | l’inscription dans les réseaux médico-sociaux et l’utilisation de ces réseaux |
|  | les comportements de santé de la famille |
|  | le développement de l’enfant |

De manière à rendre le protocole plus facile à appréhender dans ses objectifs, trois grandes catégories seront rassemblées, afin de définir les modalités d’intervention tout au long du protocole :

1. Santé mentale et physique de l’enfant
   1. réduire l’incidence des troubles de santé mentale et notamment des troubles externalisés à 2 ans^43^,
   2. favoriser la sécurité de l’attachement^69^,
   3. favoriser le développement de l’enfant^104^.
2. Santé mentale et physique de la mère
   1. réduire l’incidence de la dépression périnatale^51^,
   2. favoriser les compétences parentales et le sentiment de compétence parentale^105^,
   3. favoriser les comportements de santé de la famille^106^.
3. Réseaux sociaux et médico-sociaux
   1. favoriser l’utilisation d’un réseau social personnel et favoriser le soutien social perçu^99^,
   2. favoriser l’inscription dans les réseaux médico-sociaux et l’utilisation de ces réseaux^69, 107, 108^.

🡺 On qualifiera ce temps de PROTOCOLE PROACTIF

Au début de l'intervention, l’objectif principal (car déterminant) sera de créer une alliance thérapeutique avec la femme et la famille favorisant, entre autres, une utilisation optimalisée des services sanitaires et sociaux existants. L’intervenant s’appuiera sur le réseau de partenaires sur le terrain (PMI, secteurs de psychiatrie infanto-juvénile, crèches, aide sociale) afin d’assurer la continuité et la complémentarité du soutien. ainsi que sur des outils audiovisuels et ressources déjà validés et en langue française, et couramment utilisés au Québec ^101^.

Tout au long de l’étude, l’intervenant prendra soin d’impliquer l’ensemble des personnes présentes dans la famille (conjoint, parents, fratrie, …) dans ce « processus CAPEDP ».

# 6. Critères d’évaluation

## Critères de jugement principaux

- La survenue d’une dépression postnatale : le diagnostic sera défini par un score de l’*Edinburgh Post-partum Depression Scale* (EPDS) supérieur à 11.
- L’acquisition des compétences parentales : le critère de jugement sera l’échelle *Home Observation for the Measurement of the Environment* (HOME).
- La prévalence des troubles de santé mentale de l'enfant : le critère de jugement sera la *Child Behavior Checklist* (CBCL).

## Critères de jugements secondaires

- la connaissance et l’utilisation du système sanitaire, éducatif et social seront évaluées par le *Questionnaire sur les services médico-sociaux (SERVICES)*
- les connaissances sur le développement de l’enfant : le critère de jugement sera le *Knowledge of Infant Development Inventory (KIDI)*
- le réseau social et le soutien social perçu par les mères : *Questionnaire des réseaux et de soutien social (QSS)*
- le sentiment de compétence parentale : le critère de jugement sera l'*Echelle des cognitions et conduites parentales* (*PACOTIS)*
- le développement psychomoteur de l’enfant : le critère de jugement sera le *Brunet-Lézine Révisé (BL-R)*
- le retrait et les symptômes de détresse psychologique chez l'enfant à 18 mois : les critères de jugement seront l'*Echelle d’Alarme Détresse Bébé (ADBB)* et le *Questionnaire des comportements de l’ELDEQ (BEH)*
- le stress parental : le critère du jugement sera le *Parental Stress Inventory (PSI)*
- les symptômes de troubles psychologiques chez la mère : *Symptom Check-List (SCL-90)*
- l’inscription sociale de la mère en termes de formation et d’activité professionnelle : le critère de jugement sera le *Questionnaire sociodemographique de santé*
- l'alliance de travail entre intervenante et mère (pour le groupe intervention) : les critères de jugement seront le *Working Alliance Inventory (WAI)* et l'adhésion au programme
- l'attachement de la mère : *Vulnerable Attachment Style Questionnaire (VASQ)*
- l'attachement de l'enfant : *Attachement Q-Sort (AQS)*

La qualité de l’attachement de l’enfant et de la mère fera l’objet d’une étude ancillaire portant sur 100 femmes tirées au sort parmi celles participant à l’étude (50 dans chaque bras). Cette étude a comme objectif de montrer les liens entre attachement sécure et résilience aux facteurs de vulnérabilité, ce qui ne peut être fait avec les seuls VASQ et AQS. Elle permettra également de mettre en œuvre pour la première fois en France des évaluations de la qualité de l'attachement chez la mère ainsi que chez l'enfant.

- l'attachement de la mère : *Attachement Style Inventory (ASI)*
- l'attachement de l'enfant : *Situation Etrange (SE)*

## Variables mesurées

Seront utilisés les outils suivants :

| ***Critère de jugement*** | ***Outil*** | ***Acronyme*** |
| --- | --- | --- |
| *Dépression pré et post-natale,* | *Edinburgh Post Natal Depression Scale* | *EPDS* |
| *Compétences parentales* | *Home Observation Measurement of Environment* | *HOME* |
| *Troubles psychosociaux de la petite enfance.* | *Child Behaviour Checklist*  *Comportements de l’enfant* | *CBCL*  *BEH* |
| *Retrait relationnel* | *Echelle d’Alarme Détresse Bébé* | *ADBB* |
| *Attachement de l’enfant,* | *Attachement Q-Sort*  *Situation Etrange** | *AQS*  *SE* |
| *Compréhension et utilisation des réseaux médico-sociaux de la famille,* | *Questionnaire sur les services médico-sociaux* | *SERVICES* |
| *Soutien social de la famille,* | *Questionnaire des réseaux et de soutien social* | *QSS* |
| *Sentiment de compétences parentales* | *Pacotis* | *PACOTIS* |
| *Développement moteur, émotionnel, cognitif et social de l’enfant,* | *Brunet-Lézine Révisé* | *BL-R* |
| *Connaissances sur le développement de l’enfant* | *Knowledge of Infant Development Inventory* | *KIDI* |
| *Stress Parental / attitudes par rapport au soins* | *Parental Stress Inventory* | *PSI* |
| *Attachement de la mère* | *Vulnerable Attachment Style Questionnaire*  *Attachement Style Inventory* | *VASQ*  *ASI* |
| *Santé mentale de la mère* | *Symptom Check-List* | *SCL-90* |
| *Alliance de Travail (groupe intervention seulement)* | *Working Alliance Inventory* | *WAI* |

* En raison de la durée importante de passation de la *Situation Etrange* (à 12-15 mois) et du fait que, à la différence de l’ensemble des autres outils, il étudie directement le comportement de l’enfant, cet outil fera l’objet d’une recherche de consentement spécifique (voir Annexe 2 : formulaire de consentement spécifiques recueillant le consentement de la mère et du père pour les 100 familles en question et le consentement des parents des mères mineures).

Les interventions, protocolisées, seront évaluées et supervisées de façon intensive.

La chronologie des visites d’évaluation ainsi que les outils proposés à chaque temps sont détaillés en annexe 3.

**Le *Questionnaire socio-démographique et de santé***

Ce questionnaire socio-démographique est inspiré du modèle utilisé par l’étude longitudinale sur le développement des enfants du Québec (ELDEQ)^109^. Il vise à recueillir différents types d’informations – selon le moment d’évaluation - portant sur :

- - le ménage (composition de la famille, conditions de logement…).
  - les parents du nouveau-né (nombre d’années d’études, activité actuelle, revenu total du ménage, état de santé, précédentes expériences de grossesses de la mère, relation mère-enfant, les rôles parentaux …).
  - l’enfant (sommeil, santé, tempérament de l’enfant (en demandant aux parents d’évaluer le degré de difficulté que présente l’enfant, le développement moteur et social)
  - les grands-parents (en vie/décédés, âge).
  - la perception des répondants de leur situation socio-économique.
  - l’allaitement.
  - les habitudes de vie de la famille.
  - la consommation de tabac, d’alcool et de drogues de la mère.
  - Attitudes parentales coercitives (dont maltraitance) éventuelles.

L’utilisation de cet outil nous permettra de comparer nos données à celles recueillies au Canada dans le cadre du projet d’intervention précoce mis en place simultanément à Québec et à celles de l’étude ELDEQ 1998-2002.

**L’*Edinburgh Post-partum Depression Scale* (EPDS) ^45^**

Cet auto-questionnaire est le plus utilisé pour le dépistage de la dépression en post-partum. Il a été conçu spécialement pour cette période et a été traduit et validé en France^95^ comme dans de nombreux autres pays. De passation très courte (10 items, avec réponse en échelle de Likert en 4 points), l’EPDS est très fréquemment utilisée dans les études épidémiologiques^53^. Bien que spécifique au repérage de la dépression du post-partum, il a également été reconnu efficace dans le dépistage des états dépressifs en pré-partum, permettant ainsi d’observer sur un même outil l’évolution d’un état dépressif tout au long de la grossesse^110^. Un score supérieur à 11 définira le diagnostic de dépression postnatale^95^.

**La *Home Observation for the Measurement of the Environment* (HOME) ^111^**

Cette échelle américaine mesure le contexte social et environnemental de la vie du bébé chez lui. Les auteurs, Betty Caldwell et Robert Bradley, l’ont élaborée en 1979 puis révisée en 1984^112^. Elle nécessite la présence d’un ou des parents. Réalisée à domicile, les auteurs recommandent de l’utiliser à l’occasion d’une visite n’ayant pas pour unique but la passation de cette échelle, ce qui peut diminuer le caractère intrusif. Ce sera le cas dans cette étude car la visite aura aussi pour but d’évaluer les autres critères de jugement. Quatre grands thèmes sont abordés : les sorties et les visites à la maison ; les jouets et leur disponibilité ; l’organisation de la famille dans le quotidien ; la discipline et l’éducation. L’interprétation se fait suivant six dimensions : niveau d’attention accordé à l’enfant ; acceptation de l’enfant ; organisation et sorties ; matériel éducatif disponible ; investissement de l’enfant ; occasions de variété. L’échelle a été traduite et utilisée en France, notamment dans le cadre d’une étude européenne, en 2004^113^.

**La *Child Behavior Checklist 1½-5* (CBCL 1½-5) ^114^**

La *Child Behavior Checklist* est un des instruments de mesure de la psychopathologie infantile les plus utilisés dans la recherche et la pratique clinique. Il est destiné à fournir une description standardisée des troubles émotionnels et/ou comportementaux chez les enfants âgés de 1,5 à 16 ans. Une version française, *« la liste des comportements pour les enfants »*, a été développée et utilisée pour un sous-échantillon de garçons consultants âgés de 6 à 11 ans^114^. Les résultats obtenus par Fombonne confirment la validité discriminante de la version française et soulignent la stabilité interculturelle de la plupart des regroupements dérivés du CBCL. Il permet l'expression de scores et de percentiles pour les troubles externalisés et internalisés, l'efficience sociale et scolaire et les activités, la compétence globale et 8 syndromes comportementaux : comportement agressif, anxieux-déprimé, troubles de l'attention, comportement opposant, difficultés sociales, plaintes somatiques, troubles de la pensée, comportement évitant/déprimé. Il comprend aussi 6 échelles établies en relation avec le DSM permettant une certaine correspondance diagnostique avec ce dernier : troubles affectifs, anxieux, somatisation, hyperactivité/troubles de l'attention, comportement opposant et trouble des conduites. Les parents répondent aux items selon trois valeurs, de «faux» (0) à «tout à fait ou souvent vrai» (2).

**Le questionnaire des comportements de l’ELDEQ (BEH)^115^**

Cette échelle investigue l’ensemble des comportements psychosociaux pouvant constituer des sources de souffrance pour l’enfant et sa famille à 18 mois. Les parents de l’enfant sont invités à évaluer la pertinence d’un comportement décrit pour leur propre enfant en cotant les items selon qu’ils lui correspondent ou non. Ce questionnaire a été utilisé pour les études épidémiologiques ELNEJ et ELDEQ au Canada. Son utilisation permettra de plus de comparer notre échantillon à 18 mois à l’échantillon de l’étude réalisée à Québec.

**L’*Echelle Alarme Détresse Bébé* (ADBB)^116^**

Cette courte échelle en 8 items élaborée par Guédeney**^116^** a été construite et testée en situation clinique sur une population d’enfants entre 2 et 24 mois en vue de contribuer au repérage précoce du retrait relationnel. Les 8 items se cotent sur une échelle en 4 points.

**L’*Attachment Q-Sort* (AQS)** ^117^

Waters & Deane ont conçu un « Q-Sort » destiné à décrire la relation d'attachement entre enfant et parent et utilisable de 12 mois à 5 ans. Il comporte une centaine d'items, définis à partir de la conceptualisation théorique de Bowlby^60^. Chacun de ces items est transcrit sur une carte, qui, après une procédure de classification (en fonction de la correspondance de l’item au comportement de l’enfant), obtient alors un score (de 1 à 9) traduisant le degré de pertinence du comportement décrit, selon le point de vue de l'observateur (procédure dite « Q-Sort »). L'analyse des données revient simplement à corréler les scores du Q-Sort de l'observateur au prototype *sécure*. Le coefficient obtenu pourra être utilisé comme coefficient de sécurité. Le Q-sort comporte aussi une échelle de dépendance, particulièrement utile à mesurer comme facteur de risque de troubles ultérieurs du comportement. Le Q-Sort représente une mesure écologique de la sécurité de l’attachement et du comportement de base sûre, et est considéré actuellement comme un « gold standard »^118^. Le Q-sort, utilisé par l’évaluateur nécessite un temps d’observation à domicile (qui peut être un temps consacré à d’autres outils d’évaluation) et 30 minutes de cotation (ultérieure). Cette recherche constituera l’opportunité de valider l’instrument sur un échantillon français.

**La *Situation Etrange* (SE) ^88^**

La situation étrange est une procédure de laboratoire à l'intention des enfants âgés entre 12 et 18 mois qui implique leur mère et une personne étrangère. Elle est composée d'une introduction de 30 secondes et de courts épisodes de 3 minutes dans lesquels l'enfant est soit seul, soit avec sa mère, soit avec sa mère et une personne étrangère (travaillant pour cette étude), soit seulement avec cette personne qu'il ne connaît pas. Il s’agit d’observer comme l'enfant s'adapte à ces différentes situations.

**Le *Questionnaire sur les services***

Ce questionnaire vise à évaluer le nombre et la qualité des contacts de la famille (mère, bébé ou autre membre) avec les systèmes médico-sociaux. Sont évalués le nombre de visites, la satisfaction des services reçus et, si le service n’a pas été utilisé, la connaissance des modalités d’utilisation de ces services. Ce questionnaire est créé pour cette recherche.

**Le *Questionnaire des réseaux et de soutien social* (QSS)**

Le QSS porte sur la perception de soutien et la satisfaction avec le soutien reçu. Six différentes questions portant sur des situations nécessitant un degré de soutien sont posées. Le participant nomme toutes les personnes perçues comme pouvant être aidantes dans ces diverses circonstances. Ensuite, elle nomme le degré de satisfaction qu'elle a de son réseau de soutien. Il s'agit d'une mesure très souvent utilisée dans les travaux portant sur la notion de soutien social. Il sera construit pour la recherche à partir des questionnaires de Sarason^119^, de Barrera^120^ et de Vaux^121^.

**L'*échelle des cognitions et conduites parentales* (Pacotis)^122^**

Cette échelle comporte 26 items évaluant les perceptions des parents sur leurs comportements à l'égard de leur enfant, témoignant de leur sentiment de compétence et de leur investissement auprès de leur nouveau-né. Elle comprend quatre sous échelles : deux portant sur le sentiment de compétence et le sentiment d’impact sur le développement de l’enfant (échelles de croyances) et deux sur les comportements surprotecteurs et/ou hostiles des parents.

**Le *Brunet-Lézine* (BL-R) ^123^**

Cet outil est adapté aux enfants de 0 à 5 ans. Il mesure un niveau d'élaboration du comportement pour évaluer la vitesse de développement psychomoteur. L'analyse des résultats permet le calcul d'âges de Quotients de Développement (QD). Quatre sont partiels et concernent les quatre domaines évalués (nommés ci-dessous), le dernier est global. Il possède également la particularité d'établir des estimations par paliers de un mois jusqu'à l'âge d'un an, puis des paliers de trois mois ensuite.

Les quatre secteurs de développement sont :

- le contrôle postural et la motricité (noté P) : épreuves de locomotion et de contrôle postural de l'enfant en position dorsale, ventrale, assise et debout ;
- la coordination oculomotrice et les conduites d'adaptation par rapport aux objets (noté C) : étude de la préhension, du comportement de l'enfant face aux objets, de l'imitation de gestes ;
- le langage (noté L) : étude du langage dans ses fonctions de compréhension et d'expression ;
- les relations sociales (noté S) : étude des relations sociales englobant l'adaptation à différentes situations sociales et quotidiennes, la prise de conscience de soi et celle d'autrui.

Ce test dure environ 30 minutes. Il se compose de 17 niveaux d'âge entre 1 et 30 mois. Le score obtenu permet d'établir un Quotient de Développement (QD). Il n'y a pas de mesure directe de l'intelligence, seulement une évaluation de la vitesse de développement psychomoteur.

**Le *Knowledge of Infant Development Inventory* (KIDI)^124^**

Cette mesure est souvent utilisée pour documenter les connaissances sur le développement dans des contextes d'intervention ou de risque social. Elle contient 48 items (à coter selon que le répondant est « d’accord » ou « pas d’accord ») portant sur la période du nourrisson, d’âge préscolaire et scolaire, évaluant les connaissances des parents sur un ensemble de thématiques : développement psychosocial normatif de l’enfant, développement somatique, santé et comportements de sécurité à tenir. Les réponses sont cotées soit comme « correctes » (1) soit comme « incorrectes » (0). Cet outil a été largement utilisé dans les études internationales et les protocoles d’intervention préventive à domicile^125^.

**Le *Parental Stress Inventory* (PSI) ^126^**

Le Parental Stress Inventory est un instrument destiné au dépistage des attitudes parentales pouvant constituer des facteurs de risque de troubles émotionnels et du développement chez le jeune enfant. L’utilisation du PSI est généralement recommandée pour les cliniciens de première ligne, dans une visée de dépistage préventif. Son utilisation est recommandée par le *Report of the Surgeon General's Conference on Children's Mental Health* (2001).

**Le *Vulnerable Attachment Style Questionnaire*** **(VASQ)^127^**

Ce questionnaire élaboré par A.Bifulco vise à décrire les stratégies d’attachement d’un individu adulte par le biais d’une courte évaluation par un auto-questionnaire à 13 items et dont les scores sont projetés sur deux axes : l’un dit de « sécurité/insécurité », l’autre de « proximité/distance ». L’utilisation de cet outil constituera une occasion de le traduire et de le valider en France.

**L’*Attachment Style Inventory*, version courte (ASI) ^128^**

L'ASI est un entretien structuré proposé par A.Bifulco (2002). Il permet l'évaluation du soutien social et du style d'attachement, en 15 à 30 minutes. Le soutien social est évalué avec le partenaire proche (*Very Close Other - VCO*). Le style d'attachement est sécure ou insécure, et les insécures sont de 4 types (emmêlé, craintif, coléreux/évitant, retrait). L'outil est utilisable en épidémiologie, a été validé en Français et a été utilisé pendant la grossesse. Il permettrait dans notre étude de voir l'effet de l'intervention sur le soutien social. Il est aussi une mesure de la sécurité de l'attachement.

**La *Symptom Check-list* (SCL-90) ^129^**

LA SCL-90 est un inventaire de symptômes psychiatriques composé de 90 items et mesurant l’ampleur des symptômes actuellement expérimentés par l'individu. Il se compose de 9 sous-échelles et de 3 indices globaux de sévérité. La SCL est utilisé dans de nombreuses études et présente régulièrement d'excellents indices de validité et de fidélité.

**Le** ***Working Alliance Inventory* (WAI) ^130^**

Cette échelle évalue l’alliance thérapeutique (validation française par Guédeney et coll. ^131^). Les items du WAI sont cotés sur une échelle de Likert, de 1 à 7. Cet outil permet d’évaluer les bénéfices ressentis dans le cadre d’une interaction de type d’aide.

# 7. Schéma et conduite de la recherche

## Signature du consentement

Le consentement de la mère sera recueilli par le gynécologue obstétricien entre 12 et 27 semaines d’aménorrhée après qu’une information sur l’étude aura été donnée par un psychologue. (Annexe 1a)

Après la naissance de l’enfant, le consentement des deux titulaires de l’exercice de l’autorité parentale sera recherché pour la participation de l’enfant à la recherche. (Annexe 1b)

Un consentement spécifique et prévu pour la participation à l’étude ancillaire « Attachement mère-enfant »

Pour les femmes mineures, le consentement devra être aussi signé par les deux détenteurs de l’autorité parentale, sauf dans le cas de femmes mineures émancipées (femmes mariées ou déclarées émancipées par le juge des tutelles). (Annexe 1d).

Dans le cas où une femme accepterait la participation à l’étude, mais le co-détenteur de l’autorité parentale la refuserait pour son enfant, la femme serait suivie pour l’évaluation du critère de jugement sur la dépression post-natale jusqu’au 3^e^ mois post-partum

## Suivi des sujets

L’évaluation se fera au cours d’un entretien spécifique avec un membre de l’équipe de recherche, à l’inclusion, puis à 3, 6, 12, 18 et 24 mois après l’accouchement. Le contenu des visites d'évaluation avec les outils utilisés est décrit en Annexe 3. Par ailleurs, des données médicales périnatales seront recueillies à partir du dossier médical. Après l’accouchement, pour limiter les risques de perdus de vue, un contact téléphonique à la mère permettra notamment de savoir si elles ont des projets en termes de changement d’adresse.

Les centres investigateurs sont au nombre de six (maternités de Bichat, Louis Mourier, Beaujon, Robert Debré, Saint-Antoine, Pitié-Salpêtrière).

Le programme d'interventions pour les femmes incluses dans le groupe intervention renforcée (voir ci-dessus §5 *Intervention évaluée)* commencera dès que le premier entretien d'évaluation aura eu lieu. L’intervenant s’appuiera sur le réseau de partenaires sur le terrain (PMI, secteurs de psychiatrie infanto-juvénile, crèches, aide sociale) afin d’assurer la continuité et la complémentarité du soutien.

## Supervision des intervenants et des évaluateurs

Dans les études similaires au niveau international, il a été montré que la supervision et le contrôle du respect des procédures influencent le risque de perdus de vue de façon significative^74^ ; par ailleurs, le nombre d’heures de supervision influence l’observance de façon significative^100^. Dans l'étude actuelle, la supervision des intervenants et des évaluateurs sera assurée par les psychiatres cliniciens investigateurs dans cette recherche ; le respect du protocole de l’intervention, en particulier la réalité et la régularité des VAD, sera contrôlé par le chef de projet. La supervision sera hebdomadaire (durant une heure) pour chaque intervenant avec des réunions de groupe toutes les deux semaines. Les évaluateurs seront supervisés par le chef de projet.

## L’attitude à avoir en cas de situation d’urgence et le monitoring attenant

Une attention particulière sera apportée aux cas où un intervenant ou un évaluateur constaterait une situation d’urgence où il estimerait qu’il existe un risque pour l’enfant ou la mère (maladie, maltraitance, négligence…), que cette situation ou risque soit lié ou non à l’étude. Dans ces cas, l’intervenant contactera immédiatement le superviseur (une permanence téléphonique sera assurée). Tous deux décideront de la procédure à suivre. Si des mesures d’urgence sont prises, le chef de projet et l’investigateur seront informés. L’assistante de recherche clinique aura des contacts réguliers avec les superviseurs des intervenants et évaluateurs pour s’assurer que, à chaque fois qu’il y a de telles situations, elles ont été gérées correctement et que le promoteur a été informé.

## Comité scientifique

Il sera constitué notamment des initiateurs cliniciens du projet, du biostatisticien en charge du projet, des représentants du promoteur AP-HP à la DRRC et dans l’URC nommés pour cette recherche.

Il définira l'organisation générale et le déroulement de la recherche et coordonnera les informations.

Il décidera en cours de recherche des conduites à tenir dans les cas imprévus, surveillera le déroulement de la recherche en particulier sur le plan de la tolérance et des évènements indésirables.

## Durée totale prévisionnelle de la recherche

La période d’inclusion sera de 21 mois et le suivi de 2 ans et 5 mois. L’étude durera donc 50 mois.

## Règles d’arrêt

La décision d’arrêter l’étude sera prise par le comité scientifique. La convocation d’une réunion extraordinaire pourra avoir lieu à la demande de l’investigateur principal, du méthodologiste, ou du promoteur dans l’hypothèse où des évènements indésirables graves ou des résultats pouvant remettre en cause l’existence du protocole surviendraient.

Sera aussi considéré comme pouvant motiver l’arrêt de l’étude le recrutement insuffisant : si à 50% de la durée prévisionnelle des inclusions malgré l’aide de l’Unité de recherche clinique (URC) auprès des investigateurs et les actions entreprises pour remédier à l’échec des inclusions, moins de 30% des sujets ont été inclus, la DRRC est susceptible de décider de l’arrêt des inclusions, voire selon le contexte de l’arrêt prématuré de la recherche.

# 8. Nombre de personnes et justification

Cette étude comporte trois objectifs principaux : la diminution de la prévalence des troubles psychosociaux chez les enfants à 2 ans, l’acquisition de compétences parentales aux 2 ans de l’enfant et la diminution de la prévalence des dépressions postnatales chez la mère, au 3^e^ mois post-partum.

Pour mesurer la survenue d’une dépression postnatale, le diagnostic sera défini par un score de l’*Edinburgh Post-partum Depression Scale* (EPDS) supérieur à 11^95^. D’après la littérature, l’incidence de la dépression postnatale en population générale est de 13 à 15% Dans la population de cette étude, constituée de jeunes femmes, l’incidence attendue est plus élevée, de l’ordre de 20%. Afin de montrer une différence de 10 points entre les deux groupes (20% de dépression postnatale à 6 mois dans le groupe témoin et 10% dans le groupe intervention), il est nécessaire d’inclure au minimum 199 patientes par groupe pour un test bilatéral avec un risque alpha de 5% et une puissance de 80%, soit 398 femmes au total.

Pour mesurer l’acquisition des compétences parentales, le critère de jugement sera l’échelle *Home Observation for the Measurement of the Environment* (HOME). D’après une publication portant sur une population proche de celle de notre étude^96^, le score attendu dans le groupe soins usuels est de l’ordre de 25.5 et l’écart-type de 4.3. Afin de montrer une augmentation de ce score de 2 points dans le bras intervention, il est nécessaire d’inclure au minimum 99 personnes par groupe pour un test bilatéral avec un risque alpha de 5% et une puissance de 90%, soit 198 sujets au total.

Pour mesurer la prévalence des troubles psychosociaux, la *Child Behavior Checklist* (CBCL) sera utilisée. Nous nous appuierons donc essentiellement sur l’échelle de troubles externalisés, troubles les plus discriminants en termes de santé mentale à 2 ans. Dans une population témoin similaire^97^, le score moyen à cette échelle est 48,5 et l’écart type 8,95. Avec un test bilatéral au risque alpha de 5% et une puissance à 90%, 189 sujets par bras seront nécessaires pour pouvoir mettre en évidence une différence de 2 points, soit 378 sujets au total.

Pour pouvoir répondre aux trois objectifs principaux, nous prévoyons donc d’inclure 440 femmes, cet effectif permettant de tenir compte des possibles perdus de vue à 2 ans.

# 9. Analyse statistique

## Gestion des données et analyse statistique

##### Stratégie d’analyse des données collectées

L’analyse sera faite en intention de traiter : d’une part les sujets seront analysés selon leur groupe d’intervention et d’autre part les analyses principales se feront sur la totalité des sujets, y compris s’il existe des données manquantes. Le traitement des données manquantes fera l’objet d’une attention particulière, car l’hypothèse de données manquantes aléatoirement est peu réaliste. Deux approches seront retenues : en analyse principale, la méthode du LOCF (*Last Observation Carried Forward*) sera appliquée et, dans un second temps, nous effectuerons une analyse de sensibilité pour étudier la robustesse de nos résultats. Diverses hypothèses de lien entre le mécanisme de perdu de vue et les scores étudiés seront explorées, l’inférence se faisant par imputation multiple.

**Analyse principale** :

- Dépression postnatale : le pourcentage de femmes ayant une dépression postnatale (score EPDS > 11) à 3 et 6 mois sera comparé entre les deux groupes par un test de Chi2. Si les conditions de validité ne sont pas remplies (faible prévalence), un test exact de Fisher sera préféré.
- Compétences parentales : en première analyse, on comparera le score moyen HOME mesuré à 24 mois entre les 2 bras par un test de Student. La population d’analyse sera la totalité des femmes incluses dans l’étude. Pour les femmes perdues de vue, la dernière valeur mesurée sera utilisée (méthode LOCF). Une sous-analyse sur les femmes suivies à 24 mois sera également réalisée (analyse des *completers*). En seconde analyse, on comparera les mesures répétées de ce score au cours du temps. Une Anova sur données répétées (Manova) sera réalisée afin d’une part de déterminer des profils d’évolution du score de compétence au cours du temps et d’autre part de comparer ces scores entre les 2 groupes aux 2 temps.
- Troubles de santé mentale et du comportement : en première analyse, on comparera le score moyen CBCL mesuré chez les enfants à 24 mois entre les 2 bras par un test de Student. La population d’analyse sera la totalité des enfants dont les mères ont été incluses dans l’étude. Pour les femmes/enfants perdus de vue, la méthode d’imputation multiple sera utilisée. Une sous-analyse sur les enfants suivis à 24 mois sera également réalisée (analyse dite des *completers*).

**Analyses secondaires**

- Les mesures de l’Attachment Q-sort sont des coefficients de corrélation qui seront comparés par des tests de Student (équivalant à comparer les pentes d’une régression linéaire).
- Les autres scores seront comparés par des tests t de Student.
- Le questionnaire socio-démographique et de santé, le questionnaire des services feront l’objet d’une analyse descriptive et les scores des sous-échelles seront comparés par des tests de Student à 6 ou 24 mois.
- Dans le groupe intervention, l’adhésion au programme de prévention sera évaluée par un taux d’adéquation et son intervalle de confiance. Une sous-analyse des critères principaux sur les femmes présentant un taux d’adéquation de plus de 80% sera réalisée pour déterminer l’impact de l’adhésion sur les résultats.

Tous les tests seront bilatéraux et effectués au seuil 5%. Des calculs de puissance a posteriori seront effectués.

##### Justification des tests statistiques

Les scores étudiés ont suffisamment de modalités pour être considérés comme des variables continues. C’est d’ailleurs le traitement qu’ils reçoivent dans la littérature. Du fait de la taille d’échantillon importante, les tests paramétriques sont adaptés même si la distribution du score s’éloigne de la loi normale.

##### Responsable de l’analyse des données et logiciels de travail

Toutes les analyses statistiques seront effectuées avec le logiciel SAS v9.1 sous la responsabilité du Dr F.Tubach, du département d’Epidémiologie Biostatistique et Recherche Clinique de l’hôpital Bichat. Une déclaration de l’étude sera faite auprès de la Commission Informatique et Libertés (CNIL).

# 10. Aspects logistiques légaux et généraux

**Rôle du promoteur**

Il est défini par la loi 2004-806 du 9 août 2004. Dans cette recherche, l'AP-HP sera le promoteur et la Délégation Régionale à la Recherche Clinique (DRRC) qui en assure les missions réglementaires aura un rôle décisionnel.

##### Soumission du protocole au CCPPRB

En accord avec l'article L.1123-6 du Code de la Santé, le protocole de recherche sera soumis au Comité consultatif de Protection des Personnes en Recherche Biomédicale de Paris Saint-Louis, après accord du promoteur (avec l’attestation d’assurance et la quittance de droit fixe). L'avis de ce comité sera notifié dans le formulaire adressé à l’autorité compétente par le promoteur avant le démarrage de la recherche.

##### Déclaration CNIL

La loi prévoit que la déclaration du fichier informatisé des données personnelles collectées pour la recherche doit être faite avant le début effectif de la recherche.

Cette recherche nécessite **une déclaration unitaire**.

Une copie de la réponse favorable de la CNIL, sera envoyée à la DRCD dès l’obtention de cet avis.

##### Documentations de la recherche

Avant de démarrer la recherche, chaque investigateur fournira au représentant du promoteur de la recherche une copie de son **curriculum vitæ personnel daté et signé** et comportant son numéro d’inscription à l’ordre des médecins.

La version du protocole avec ses annexes sera signée conjointement par l’investigateur coordonnateur et le représentant du promoteur avant demande d’autorisation et avis du CCPPRB. Le cas échéant, le responsable scientifique sera également signataire.

A chaque nouvelle version du protocole, rendue nécessaire par des amendements et/ou demandes des autorités, un nouveau numéro de version ainsi que la date correspondante seront attribués et les mêmes signatures recueillies.

Chaque investigateur s'engagera à respecter les obligations de la loi et à mener la recherche selon les B.P.C., en respectant les termes de la déclaration d'Helsinki. Pour ce faire, un exemplaire daté et signé de **l’engagement scientifique (document type DRRC) par chaque investigateur** de chaque service clinique participant d’un centre sera remis au représentant du promoteur.

##### Contrôle de Qualité et Assurance Qualité

La recherche sera encadrée selon les procédures opératoires standard de l’AP-HP promoteur.

Le déroulement de la recherche dans les centres investigateurs et la prise en charge des sujets sera faite conformément à la déclaration d’Helsinki et les Bonnes Pratiques.

**Procédures de monitoring**

Les représentants du promoteur effectueront des visites des centres investigateurs au rythme des inclusions, dans les différents centres et au niveau de risque qui a été attribué à la recherche (risque B).

- D’abord, avant inclusions, pour une visite d‘ouverture de chaque centre avec mise en place du protocole et prise de connaissance avec les investigateurs.

- Les données d'inclusion seront monitorées à partir du dossier source de la maternité. Le monitoring pour le suivi consistera en des contacts réguliers avec les superviseurs des intervenants et des évaluateurs pour s'assurer que les évènements indésirables ont bien été transmis au promoteur.

- Lors des visites de monitoring suivantes, les cahiers d'observation seront revus au fur et à mesure de l'état d'avancement de la recherche par les ARCs représentant le promoteur. L'investigateur principal de chaque centre et les autres investigateurs qui incluent ou suivent des sujets participant à la recherche s’engagent à recevoir des représentants du promoteur nommés par l’AP-HP à intervalles réguliers.

Lors de ces visites sur site et en accord avec les Bonnes Pratiques Cliniques, les éléments suivants seront revus :

- respect du protocole de la recherche et des procédures définies,

- examen des documents source et confrontation avec les données reportées dans le cahier d’observation,

- contrôle de qualité des données recueillies dans le cahier d'observation : exactitude, données manquantes, cohérence des données, selon les règles édictées par les procédures de la DRRC.

##### Gestion des Événements Indésirables

##### Définitions

- **Evénement indésirable**

Toute manifestation nocive et non recherchée survenant chez une personne pendant une recherche, qu’elle soit considérée ou non comme liée à celle-ci.

- **Effet indésirable**

Réaction nocive et non voulue à un traitement expérimental : médicament (quelle que soit la dose administrée), dispositif, traitement chirurgical…) utilisé chez l’homme.

- **Effet ou événement indésirable grave**

Effet ou événement indésirable ayant entraîné :

- Le décès

- La mise en jeu du pronostic vital

- Une invalidité ou une incapacité importantes ou durables

- Une hospitalisation ou prolongation d’hospitalisation

- Une anomalie ou malformation congénitale

- Autre : tout effet indésirable jugé comme grave par le professionnel de santé, en particulier les événements nécessitant une intervention pour éviter l’une des conséquences notées ci-dessus, et certains résultats d’examens paracliniques.

- **Effet indésirable inattendu**

Effet indésirable dont la nature, la gravité ou l’évolution ne correspondent pas aux informations contenues dans le résumé des caractéristiques du produit, la brochure investigateur ou autre référentiel reconnu par les autorités.

- **Situations d’urgence ou constat de risques dans l’intervention à domicile**

Une attention particulière sera apportée aux cas où un intervenant ou un évaluateur constaterait une situation d’urgence ou estimerait qu’il existe un risque pour l’enfant ou la mère (maladie, maltraitance, négligence…), que cette situation ou risque soit lié à l’étude ou non. Dans ces cas, l’intervenant contactera immédiatement le superviseur (une permanence téléphonique sera assurée). Tous deux décideront de la procédure à suivre. Si des mesures d’urgence sont prises, le chef de projet et l’investigateur seront informés. L’assistante de recherche clinique aura des contacts réguliers avec les superviseurs des intervenants et évaluateurs pour s’assurer que, à chaque fois qu’il y a de telles situations, elles ont été gérées correctement et que le promoteur a été informé.

#### Obligations des investigateurs

- **Evénements indésirables non graves :**

Tout événement indésirable - non grave suivant la définition précédente - observé lors de la recherche et dans ses suites devra être reporté dans le cahier d’observation dans la section prévue à cet effet.

Un seul événement doit être reporté par item. L’événement peut correspondre à un symptôme, un diagnostic ou à un résultat d’examen complémentaire jugé significatif. Tous les éléments cliniques ou paracliniques permettant de décrire au mieux l’événement correspondant doivent être reportés.

Tout patient présentant un événement indésirable doit être suivi jusqu’à la résolution ou la stabilisation de celui-ci, et l’évolution en sera notée sur la page correspondante.

- **Evénements indésirables graves :**

Les investigateurs doivent notifier **immédiatement** au promoteur AP-HP les événements indésirables graves tels que définis ci-dessus.

**L'investigateur complète les feuillets-type événements indésirables graves (du cahier d'observation de la recherche) et les envoie à la DRRC (en précisant le nom du chef de projet en charge de la recherche) par fax au 01 44 84 17 99 et ce, dans les 48 heures** (après si possible un appel téléphonique immédiat au 01 44 84 17 23 en cas de décès ou d’une menace vitale inattendus).

Pour chaque événement indésirable grave, **l’investigateur devra émettre un avis sur le lien de causalité entre l’événement indésirable et le protocole.**

L’obtention d’informations relatives à la description et l’évaluation d’un événement indésirable peuvent ne pas être possibles dans le temps imparti pour la déclaration initiale.

Aussi, l'évolution clinique ainsi que les résultats des éventuels bilans cliniques et des examens diagnostiques et/ou de laboratoire, ou toute autre information permettant une analyse adéquate du lien de causalité seront rapportés :

- soit sur la déclaration initiale d’EIG s’ils sont immédiatement disponibles,
- soit ultérieurement et le plus rapidement possible, en renvoyant par fax la déclaration initiale d’EIG complétée (et en précisant « Follow-up » et le numéro de suivi).

Toutes les déclarations faites par les investigateurs devront identifier chaque sujet participant à l’essai par un numéro de code unique attribué à chacun d’entre eux.

En cas de décès notifié d’un sujet participant à l’essai, l’investigateur communiquera au promoteur AP-HP tous les renseignements complémentaires demandés (compte-rendu d’hospitalisation, résultats d’autopsie…).

Tout fait nouveau survenu dans la recherche ou dans le contexte de la recherche, provenant de données de la littérature ou de recherches en cours, devra être notifié au promoteur AP-HP.

Pour le cas spécifiques des participantes à la recherche dont la grossesse serait interrompue ou dont l’enfant décéderait avant la fin de la recherche, une procédure d’orientation vers les services de soutien adaptés serait mise en place immédiatement. La participante ne participerait alors plus à la recherche.

##### Déclaration des événements indésirables graves aux Autorités de Santé

Elle sera assurée par le pôle de pharmacovigilance de la DRRC, après analyse d’imputabilité de chaque cas. Toutes les suspicions d’effet indésirable grave inattendu (non présent dans le résumé des caractéristiques du produit ou dans la brochure investigateur du ou des produits utilisés dans la recherche) seront déclarées par le promoteur à l’autorité compétente dans les délais légaux.

En cas d’effet indésirable grave inattendu dû à l’un des traitements de la recherche ou à la recherche elle-même, le CCPPRB et les investigateurs de la recherche devront être informés.

##### Transcription des données dans le cahier d’observation

Toutes les informations requises par le protocole doivent être fournies dans le cahier d’observation et une explication donnée par l’investigateur pour chaque donnée manquante.

Les données devront être transférées dans les cahiers d'observation au fur et à mesure qu'elles sont obtenues qu'il s'agisse de données cliniques ou para-cliniques. Les données devront être copiées de façon nette et lisible à l'encre noire dans ces cahiers (ceci afin de faciliter la duplication et la saisie informatique).

Les données erronées dépistées sur les cahiers d'observation seront clairement barrées et les nouvelles données seront copiées sur le cahier avec les initiales et la date par le membre de l'équipe de l'investigateur qui aura fait la correction.

L'anonymat des sujets sera assuré par la mention au maximum des 3 premières lettres du nom et des 2 premières lettres du prénom du sujet sur tous les documents nécessaires à la recherche, ou par effacement par les moyens appropriés (blanc correcteur…) des données nominatives sur les copies des documents source, destinés à la documentation de la recherche.

Les données informatisées sur un fichier seront déclarées à la CNIL selon la procédure adaptée au cas.

##### Amendements au protocole de recherche

La DRRC doit être informée de tout projet de modification du protocole par l’investigateur coordonnateur. Les modifications devront être qualifiées en substantielles ou non.

Tout amendement au protocole de la recherche, devra être notifié au CCPPRB s'il entraîne des modifications substantielles, c'est-à-dire si les modifications prévues sont susceptibles, d'une manière ou d'une autre, de modifier les garanties apportées aux personnes qui se prêtent à la recherche biomédicale *(modification d’un critère d’inclusion, prolongation d’une durée d’inclusion, participation de nouveaux investigateurs…)*.

##### Extension de la recherche

Toute extension de la recherche (modification profonde du schéma thérapeutique ou des populations incluses, prolongation des traitements et ou des actes thérapeutiques non prévus initialement dans le protocole) devra être considérée comme une nouvelle recherche.

##### Responsabilité

L'Assistance Publique-Hôpitaux de Paris est le promoteur de cette recherche. En accord avec la loi sur les recherches biomédicales, elle a pris une assurance auprès de la compagnie GERLING KonZern pour toute la durée de la recherche, garantissant sa propre responsabilité civile ainsi que celle de tout intervenant (médecin ou personnel impliqué dans la réalisation de la recherche) (loi n°2004-806, Art L.1121-10 du CSP).

l'Assistance Publique - Hôpitaux de Paris se réserve le droit d'interrompre la recherche à tout moment pour des raisons médicales ou administratives; dans cette éventualité, une notification sera fournie à l'investigateur.

##### Rapport final de la recherche

Le rapport final de la recherche sera écrit en collaboration par le coordonnateur et le biostatisticien pour cette recherche. Ce rapport sera soumis à chacun des investigateurs pour avis. Une fois qu'un consensus aura été obtenu, la version finale devra être avalisée par la signature de chacun des investigateurs et adressée au promoteur dans les meilleurs délais après la fin effective de la recherche. Un rapport rédigé selon le plan de référence de l’autorité compétente doit être transmis à l’autorité compétente ainsi qu’au Comité dans un délai de un an, après la fin de la recherche, s’entendant comme la dernière visite de suivi du dernier sujet inclus. Ce délai est rapporté à 90 jours en cas d’arrêt prématuré de la recherche.

##### Publications et propriétés des données

L’AP-HP est propriétaire des données et aucune utilisation ou transmission à un tiers ne peut être effectuée sans son accord préalable.

seront premiers signataires des publications, les personnes ayant réellement participé à l’élaboration du protocole et son déroulement ainsi qu’à la rédaction des résultats.

L’Assistance Publique- Hôpitaux de Paris doit être mentionnée comme étant le promoteur de la recherche biomédicale et comme soutien financier le cas échéant. les termes « Assistance Publique- Hôpitaux de Paris » doivent apparaître dans l’adresse des auteurs.

# 11. Retombées attendues scientifiques et en termes de santé publique

Le projet CAPEDP vise à mettre en place et à évaluer - pour la première fois en France - les effets d’un programme de prévention des troubles psychosociaux et de promotion de la santé mentale. Il s'adresse donc à un problème préoccupant – et coûteux – de santé publique, ressenti par l’ensemble des professionnels de la petite enfance (éducation nationale, services médico-sociaux, services de prévention, éducateurs, psychiatrie publique…). CAPEDP est fondé sur l’hypothèse qu'une approche préventive ciblée, en diminuant la prévalence des troubles de santé mentale dès le plus jeune âge, permettra de diminuer les besoins en termes de soins curatifs. Il s’agira également de la première étude française sur l’attachement mère-bébé dans une cohorte.

Une telle intervention devrait par ailleurs permettre de réduire la fréquence des troubles de santé mentale au sens large, dans la mesure où elle favoriserait le développement harmonieux de la relation mère-enfant, augmenterait la qualité de l’attachement entre parents et enfant, diminuerait la fréquence de la dépression postnatale, et permettrait que les mères isolées utilisent mieux les dispositifs de santé existants.

Si cette intervention ciblant une population à risque démontre son efficacité, l’objectif serait de la généraliser aux prestations de droit et de contribuer ainsi à réduire les troubles de santé mentale et du comportement, notamment dans des zones urbaines sensibles.

# BIBLIOGRAPHIE

1. Shonkoff J, Phillips D. *From neurons to neighborhoods: The science of early childhood development*. Washington, DC: National Academy Press, 2000.

2. Bronfrenbrenner U. *The ecology of human development: Experiments by nature and design*. Cambridge, MA: Harvard University Press, 1979.

3. Bronfrenbrenner U. Ecology of the family as a context for human development:Research perspectives. *Developmental Psychology* 1986;22(6):723-742

4. Bowlby J. *Attachment, Vol. 3 of Attachment and loss: Loss*. New York: Basic Books, 1980.

5. Brazelton T, Cramer B. *The first relationship*. New York: Addison Wesley, 1990.

6. Young children develop in an environment of relationships. Brandeis University, Waltham, MA: National Scientific Council on the Developing Child, 2004.

7. Davis P, Cummings E. Marital Conflict and Child Adjustment: an Emotional Security Hypothesis. *Psychological Bulletin* 1994;116:387-411

8. Weinberg M, Tronick E. The impact of maternal psychiatric illness on infant development. *Journal of Clinical Psychiatry* 1998;59:53-61

9. Clery-Melin P, Kovess V, Pascal J. Agir aux racines de la violence. Paris: Ministère de la santé, de la famille et des personnes handicapées, 2003.

10. Courtois J. PJL loi de finances pour 1999. Paris: Senat, 1998.

11. OMS. Rapport mondial sur la violence et la santé. Genève, 2002.

12. Tremblay RE, Nagin DS, Seguin JR, Zoccolillo M, Zelazo PD, Boivin M, et al. Physical aggression during early childhood: trajectories and predictors. *Pediatrics* 2004;114(1):e43-50

13. NICHD. The Development of Aggressive Behaviour in Children and Young People: Implications for Social Policy, Service Provision, and Further Research, 2003.

14. Tremblay R, Nagin DS, Seguin JR, Zoccolillo M, Zelazo PD, Boivin M, et al. Physical aggression during early childhood: trajectories and predictors. *Pediatrics* 2004;114(1):43-50

15. Welniarz B. L’enfant de trois ans violent. *L'Information Psychiatrique* 1998;74(9):888-896

16. Tremblay R. The development of aggressive behavior during childhood: What have we learned in the past century? *International Journal of Behavioral Development* 2000;24(2):129-141

17. Angold A, Egger H, Erkanli A, Keeler G. Prevalence and comorbidity of psychiatric disorders in preschoolers attending a large pediatric service. *Submitted for publication* 2005

18. Bilenberg N, Petersen D, Hoerder K, Gillberg C. The prevalence of child-psychiatric disorders among 8-9-year-old children in Danish mainstream schools. *Acta Psychiatr Scand* 2005;111(1):59-67

19. Campbell S, Pierce E, Moore G, Marakowitz S, Newby K. Boy's externalizing Problems at Elementary School: Pathways from Early Behaviour Problems, Maternal Control, and Family Stress. *Dev Psychol* 1996;8:701-720

20. Lahey B, Waldman, ID. & McBurnett, K. The development of antisocial behaviour: an integrative causal model. *J Child Psychol. Psychiat.* 1999;40:669-682

21. Lavigne J, Arend R, Rosenbaum D, Binns H, Christoffel K, Gibbons R. Psychiatric disorders with onset in the preschool years: I. Stability of diagnoses. *J Am Acad Child Adolesc Psychiatry* 1998;37(12):1246-54

22. Olds D, Eckenrode J, Henderson C, Kitzman H, Powers J, Cole R, et al. Long-Term Effects of Nurse Home Visitation on Children's Criminal and Antisocial Behavior. 15-Year Follow-up of a Randomized Controlled Trial. *JAMA* 1998;280(14):1238-1244

23. Tremblay R, Côté S. The developmental origins of aggression: Where are we going? In: Hartup W, Tremblay R, Archer J, editors. *The origins of aggression*. New York: Guilford Press, 2005.

24. Shaw DS, Owens EB, Giovannelli J, Winslow EB. Infant and toddler pathways leading to early externalizing disorders. *Journal of American Academy of Child and Adolescent Psychiatry* 2001;40:36-43

25. Broidy LM, Nagin DS, Tremblay RE, Bates JE, Brame B, Dodge KA, et al. Developmental trajectories of childhood disruptive behaviors and adolescent delinquency: a six-site, cross-national study. *Dev Psychol* 2003;39(2):222-45

26. Lacourse E, Cote S, Nagin DS, Vitaro F, Brendgen M, Tremblay RE. A longitudinal-experimental approach to testing theories of antisocial behavior development. *Dev Psychopathol* 2002;14(4):909-24

27. Nagin D, Tremblay R. Parental and early childhood predictors of persistent physical aggression in boys from kindergarten to high school. *Arch Gen Psychiatry* 2001;58(4):389-94

28. Hawkins J. Predictors of Youth Violence: Office of Juvenile and Delinquency Prevention, 2000.

29. INSERM. Troubles mentaux: Dépstage et prévention chez l'enfant et l'adolescent. Expertise collective., 2004.

30. Choquet M, Ledoux S. Adolescents: Enquête nationale. Paris: Inserm, 1994.

31. Fonagy P. Prevention, The Appropriate Target of Infant Psychotherapy. *Infant Mental Health Journal* 1998;19(2):124-150

32. Beyers JM, Loeber R, Wikstrom PO, Stouthamer-Loeber M. What predicts adolescent violence in better-off neighborhoods? *J Abnorm Child Psychol* 2001;29(5):369-81

33. Sommers I, Baskin D. Factors related to female adolescent initiation into violent street crime. *Youth Society* 1994;25:468-489

34. Stouthamer-Loeber M, Loeber R, Wei E, Farrington DP, Wikstrorm PO. Risk and promotive effects in the explanation of persistent serious delinquency in boys. *J Consult Clin Psychol* 2002;70(1):111-23

35. Sameroff A. Environmental risk factors in infancy. *Pediatrics* 1998;102(5 Suppl E):1287-92

36. Belsky J, Kuang-Hua, H., & Crnic, K. Mothering, fathering and infant negativity as antecedents of boys’ externalising problems and inhibition at 3 years; Differential susceptibility to rearing experience. *Dev Psychol* 1998;10:301-319

37. Olds D, Kitzman H, Cole R, Robinson J, Sidora K, Luckey D, et al. Effects of nurse home-visiting on maternal life course and child development: age 6 follow-up results of a randomized trial. *Pediatrics* 2002;114(6):1150-9

38. Caspi A, Roberts B, Shiner R. PERSONALITY DEVELOPMENT: Stability and Change. *Annu Rev Psychol* 2005;56:453-84

39. Sampson RJ, Laub JH. Urban poverty and the family context of delinquency: a new look at structure and process in a classic study. *Child Dev* 1994;65(2 Spec No):523-40

40. Lyons-Ruth K, Melnick S. Dose-response effect of mother-infant clinical home visiting on aggressive behavior problems in kindergarten. *J Am Acad Child Adolesc Psychiatry* 2004;43(6):699-707

41. Wakschlag LS, Lahey BB, Loeber R, Green SM, Gordon RA, Leventhal BL. Maternal smoking during pregnancy and the risk of conduct disorder in boys. *Arch Gen Psychiatry* 1997;54(7):670-6

42. Nagin DS, Tremblay RE. Parental and early childhood predictors of persistent physical aggression in boys from kindergarten to high school. *Arch Gen Psychiatry* 2001;58(4):389-94

43. Tremblay RE. Prevention of injury by early socialization of aggressive behavior. *Inj Prev* 2002;8 Suppl 4:IV17-21

44. Hay DF, Pawlby S, Angold A, Harold GT, Sharp D. Pathways to violence in the children of mothers who were depressed postpartum. *Dev Psychol* 2003;39(6):1083-94

45. Cox JL, Holden JM, Sagovsky R. Detection of postnatal depression. Development of the 10-item Edinburgh Postnatal Depression Scale. *Br J Psychiatry* 1987;150:782-6

46. Guedeney N, Fermanian J, Guelfi JD, Kumar RC. The Edinburgh Postnatal Depression Scale (EPDS) and the detection of major depressive disorders in early postpartum: some concerns about false negatives. *J Affect Disord* 2000;61(1-2):107-12

47. Harnish J, Dodge K, Valente E. Mother-child interaction quality as a partial mediator of the roles of maternal depressive symptomatology and socioeconomic status in the development of child behavior problems.Conduct Problems Prevention Research Group. *Child Dev* 1995;66(3):739-53

48. Beardslee W, Versage E, Gladstone T. Children of affectively ill parents: a review of the past 10 years. *J Am Acad Child Adolesc Psychiatry* 1998;37(11):1134-41

49. Luoma I, Kaukonen P, Mantymaa M, Puura K, Tamminen T, R S. Longitudinal study of maternal depressive symptoms and child well-being. *J Am Acad Child Adolesc Psychiatry* 2001;40(12):1367-74

50. Luoma I, Kaukonen P, Mantymaa M, Puura K, Tamminen T, R S. A longitudinal study of maternal depressive symptoms, negative expectations and perceptions of child problems. *Child Psychiatry Hum Dev* 2004;35(1):37-53

51. Murray L, Cooper J. *Post partum depression and child development*. NY: The Guilford Press, 1999.

52. Beardslee WR, Gladstone TR, Wright EJ, Cooper AB. A family-based approach to the prevention of depressive symptoms in children at risk: evidence of parental and child change. *Pediatrics* 2003;112(2):e119-31

53. Chabrol H, Teissedre F, Saint-Jean M. Dépistage, prévention et traitement des dépressions du post-partum: une étude contrôlée chez 859 sujets. *L'Encephale* 2002;XXVIII:65-70

54. Chabrol H. *Les dépressions de la maternité*. Paris: PUF, 1998.

55. Widom C. The cycle of violence. *Science* 1989;244(4901):160-6

56. Eckenrode J, Zielinski D, Smith E, Marcynyszyn L, Henderson C, Kitzman H, et al. Child maltreatment and the early onset of problem behaviors: can a program of nurse home visitation break the link. *Dev Psychol* 2001;13(4):873-90

57. NYVPRC. Depression: US Department of Health and Human Services, 2001.

58. Dionne G, Tremblay R, Boivin M, Laplante D, Perusse D. Physical Agression and Expressive Vocabulary in 19-month-old twins. *Dev Psychol* 2003;39(2):261-73

59. Shaw D, Vondra J. Infant attachment security and maternal predictors of early behavior problems: a longitudinal study of low income families. *Journal of Abnormal Child Psychology* 1995;23:335-357

60. Bowlby J. *Attachment, Vol. 1 of Attachment and loss*. London: Hogarth Press, 1969.

61. Retz W, Retz-Junginger P, Supprian T, Thome J, Rosler M. Association of serotonin transporter promoter gene polymorphism with violence: relation with personality disorders, impulsivity, and childhood ADHD psychopathology. *Behav Sci Law* 2004;22(3):415-25

62. Hudziak J, van Beijsterveldt C, Bartels M, Rietveld M, Rettew D, Derks E, et al. Individual differences in aggression: genetic analyses by age, gender, and informant in 3-, 7-, and 10-year-old Dutch twins. *Behav Genet* 2003;33(5):575-89

63. Eley T, Lichtenstein P, Moffitt T. A longitudinal behavioral genetic analysis of the etiology of aggressive and nonaggressive antisocial behavior. *Dev Psychopathol* 2003;15(2):383-402

64. Cadoret R, Langbehn D, Caspers K, Troughton E, Yucuis R, Sandhu H, et al. Associations of the serotonin transporter promoter polymorphism with aggressivity, attention deficit, and conduct disorder in an adoptee population. *Compr Psychiatry* 2003;44(2):88-101

65. Caspi A, McClay J, Moffitt T, Mill J, Martin J, Craig I, et al. Role of Genotype in the Cycle of Violence in Maltreated Children. *Science* 2002;297(5582):851-4

66. Mazet P, Stoleru S. *Psychopathologie du nourrisson et du jeune enfant*. Paris: Masson, 2003.

67. Olds DL, Henderson CR, Jr., Chamberlin R, Tatelbaum R. Preventing child abuse and neglect: a randomized trial of nurse home visitation. *Pediatrics* 1986;78(1):65-78

68. Olds DL. Prenatal and infancy home visiting by nurses: from randomized trials to community replication. *Prev Sci* 2002;3(3):153-72

69. Olds D, Henderson CR, Jr., Cole R, Eckenrode J, Kitzman H, Luckey D, et al. Long-term effects of nurse home visitation on children's criminal and antisocial behavior: 15-year follow-up of a randomized controlled trial. *Jama* 1998;280(14):1238-44

70. Kitzman H, Olds DL, Henderson CR, Jr., Hanks C, Cole R, Tatelbaum R, et al. Effect of prenatal and infancy home visitation by nurses on pregnancy outcomes, childhood injuries, and repeated childbearing. A randomized controlled trial. *Jama* 1997;278(8):644-52

71. Olds D, Robinson J, O'Brien R, Luckey D, Pettitt L, Henderson C, et al. Home Visiting by Paraprofessionals and by Nurses: A Randomized, Controlled Trial. *Pediatrics* 2002;110:486-496

72. Ross M. Starting Well in Glasgow. Promotion de la santé mentale chez le jeune enfant; 2005; Paris: MGEN.

73. Tremblay RE, Schaal B. Physically aggressive boys from age 6 to 12 years. Their biopsychosocial status at puberty. *Ann N Y Acad Sci* 1996;794:192-207

74. Davis H, Ispanovic V, Puura K, Tsiantis J, Turenen M, Paradisiotou A. Comment évaluer la promotion de la santé? Premiers résultats du Projet européen de promotion précoce de la santé. In: Haddad A, Guedeney A, Greacen T, editors. *Santé mentale du jeune enfant: prévenir et intervenir*. Ramonville: Eres, 2004.

75. Eisen A. Survey of neighborhood-based, comprehensive community empowerment initiatives. *Health Education Quaterly* 1994;21(2):235-252

76. Zimmerman M. Taking aim on empowerment research: On the distinction between individual and psychological concepts. *Américan Journal Psychology* 1990;18:169-177

77. Kieffer C. Citizen empowerment: A developmental perspectives. In: Rappaport J, Hess R, Swift C, editors. *Studies in empowerment: Steps toward understanding and action*. New York: Hayworth Press, 1984.

78. Rappaport J. Terms of empowerment/exemplars of prevention: toward a theory for community psychology. *American Journal of Community Psychology* 1987;15:121-48

79. Katz R. Empowerment and Synergy: Expanding the community's healing ressources. *Prevention in Human Services* 1984;3:201-26

80. Gibson C. A concept analysis of empowerment. *Journal of Advanced Nursing* 1991;16:354-361

81. Hawley-McWhirter E, MC WHIRTER E. EicJoCD, 69. 222-227. Empowerment in counseling. *Journal of Counseling & Development* 1991;69:222-7

82. Olds D. The nurse-family partnership: an evidence-based preventive intervention. *Infant Mental Health Journal* 2006;27(1):5-25

83. Bowlby J. *Attachment, Vol. 1 of Attachment and loss: Attachment*. New York: Basic Books, 1969.

84. Bowlby J. *Attachment, Vol. 2 of Attachment and loss: Separation*. New York: Basic Books, 1973.

85. Ainsworth M. Attachments beyond infancy. *American Psychologist* 1989;44:709-716

86. Grusec J, Lytton H. *Social development: History, theory, and research*. New York: Springer-Verlag, 1988.

87. Turner P. Relations between attachment, gender, and behavior with peers in the preschool. *Child Dev* 1991;62:1475-1488

88. Ainsworth M, Blehar M, Waters E, Wall S. *Patterns of attachment: A psychological study of the strange situation*. Hillsdale: Erlbaum, 1978.

89. Sroufe L, Egeland B, Kreutzer T. The fate of early experience following developmental change: longitudinal approaches to individual adaptation in childhood. *Child Development* 1990;61:1363-73

90. Grossmann K, Grossmann K. Attachment quality as an organizer of emotional and behavioral responses in a longitudinal perspective. In: Stevenson-Hinde J, Parkes C, editors. *Attachment across the life circle*. London: Routledge, 1991:93-114.

91. George C, Kaplan N, Main M. The Adult Attachment Interview: University of California at Berkeley, 1985.

92. Guédeney N, Guédeney A. *L'attachement: concepts et applications*. Paris: Masson, 2004.

93. Olds DL, Eckenrode J, Henderson CR, Jr., Kitzman H, Powers J, Cole R, et al. Long-term effects of home visitation on maternal life course and child abuse and neglect. Fifteen-year follow-up of a randomized trial. *Jama* 1997;278(8):637-43

94. Cox AD. Befriending young mothers. *Br J Psychiatry* 1993;163:6-18

95. Guédeney N, Fermanian J, Guelfi J, Delour M. Premiers résultats de la traduction de l'edinburgh post-natal depression scale sur une population parisienne. *Devenir* 1995;7(2):69-92

96. Armstrong KL, Fraser JA, Dadds MR, Morris J. Promoting secure attachment, maternal mood and child health in a vulnerable population: a randomized controlled trial. *J Paediatr Child Health* 2000;36(6):555-62

97. Mäntymaa M, Puura K, Luoma I, Salmelin R, Tamminen T. Early mother–infant interaction, parental mental health and symptoms of behavioral and emotional problems in toddlers. *Infant Behavior and Development* 2004;27(2):134-149

98. Olds D, Henderson CR, Jr., Kitzman H, Cole R. Effects of prenatal and infancy nurse home visitation on surveillance of child maltreatment. *Pediatrics* 1995;95(3):365-72

99. Olds DL. Home visitation for pregnant women and parents of young children. *Am J Dis Child* 1992;146(6):704-8

100. McGuigan WM, Katzev AR, Pratt CC. Multi-level determinants of retention in a home-visiting child abuse prevention program. *Child Abuse Negl* 2003;27(4):363-80

101. Japel C, Cote S, Tremblay R. La qualité des services à la petite enfance: Résultats de l'Etude longitudinale sur le développement des enfants du Québec (ELDEQ): Institut de recherche en politiques publiques, 2004.

102. Olds D, Hill P, Robinson J, Song N, Little C. Update on home visiting for pregnant women and parents of young children. *Curr Probl Pediatr* 2000;30(4):107-41

103. Davis H, Tsiantis J. Promoting Children's Mental Health: the European Early Promotion Project (EEPP). *International Journal of Mental Health Promotion* 2005;7(1):4-16

104. Olds DL, Kitzman H, Cole R, Robinson J, Sidora K, Luckey DW, et al. Effects of nurse home-visiting on maternal life course and child development: age 6 follow-up results of a randomized trial. *Pediatrics* 2004;114(6):1550-9

105. Kitzman H, Olds DL, Sidora K, Henderson CR Jr, Hanks C, Cole R, et al. Enduring effects of nurse home visitation on maternal life course: a 3-year follow-up of a randomized trial. *JAMA* 2000;283(15):1983-9

106. Fiscella K, Kitzman HJ, Cole RE, Sidora KJ, Olds D. Does child abuse predict adolescent pregnancy? *Pediatrics* 1998;101(4 Pt 1):620-4

107. Schweinhart J, Weikart D. High/Scope Perry Preschool Program outcomes. In: MccCord J, Tremblay R, editors. *Preventing antisocial behavior: Interventions from birth through adolescence*. New York: Guilford Press, 1992:67-86.

108. Campbell FA, Ramey CT. Effects of early intervention on intellectual and academic achievement: a follow-up study of children from low-income families. *Child Dev* 1994;65(2 Spec No):684-98

109. Thibault J, Jetté M, Derosiers H. Aspects conceptuels et opérationnels, section I – Conception de la phase I de l’ÉLDEQ, instruments et déroulement. *Étude longitudinale du développement des enfants du Québec (ÉLDEQ 1998-2002)*. Québec: Institut de la statistique du Québec, 2001.

110. Murray L, Cox J. Identifying depression during pregnancy with the EPDS. *Journal of Reproductive and Infant psychology* 1990;8:99-107

111. Bradley RH, Caldwell BM. Home observation for measurement of the environment: a revision of the preschool scale. *Am J Ment Defic* 1979;84(3):235-44

112. Caldwell B, Bradley R. *Home Observation for Measurement of the Environment*. Little Rock: University of Arkansas, 1984.

113. Gunning M, Conroy S, Valoriani V, Figueiredo B, Kammerer MH, Muzik M, et al. Measurement of mother-infant interactions and the home environment in a European setting: preliminary results from a cross-cultural study. *Br J Psychiatry Suppl* 2004;46:s38-44

114. Fombonne E. The Chartres Study: I. Prevalence of psychiatric disorders among French school-age children. *Br J Psychiatry* 1994;164(1):69-79

115. L'Etude longitudinale du développement des enfants du Québec, ELDEQ 1998-2002. Québec: Institut de la statistique, 2002.

116. Guédeney A, Charron J, Delour M, Fermanian J. L'évaluation du comportement de retrait relationnel du jeune enfant lors de l'examen pédiatrique par l'échelle d'alarme détresse bébé (ADBB). *Psychiatrie de l'enfant* 2001;XLIV(1):211-231

117. Waters E, Deane K. Defining and assessing individual differences in attachment relationships: Q-methodology and the organization of behavior in infancy and early childhood. In: Bretherton I, Waters E, editors. *Growing points of attachment theory and research*: Monographs of the Society for Research in Child Development, 1985:41-103.

118. van IJzendoorn M, Verejken C, MJ B-K, Riksen Walraven J. Assessing attachment security with the attachment Q sort.: meta analytic evidence for the validity of the observer AQS. *Child development* 2004;75(4)

119. Sarason I, Levine H, Basham R, Sarason B. Assessing social support: the social support questionnaire. *Journal of personality and social psychology* 1983;50:1220-1225

120. Barrera M. Distinctions Between Social Support Concepts, Measures, and Models. *American Journal of Community Psychology* 1986;14(4):413-445

121. Vaux A. *Social Support. Theory, Research, and Intervention*. New York: Praeger, 1988.

122. Boivin M, Pérusse D, Dionne G, Saysset V, Zoccolillo M, Tarabulsy G, et al. The genetic-environmental etiology of parent's perceptions and self-assessed behaviors toward their 5-month-old infants in a large twin and singleton sample. *Journal of Child Psychology and Psychiatry* 2005;43(5)

123. Brunet O, Lezine I. *Le développement psychologique de la première enfance. Présentation d'une échelle française pour examen des touts petits*. Paris: PUF, 1965.

124. McPhee. *Knowledge of infant development inventory: Human development and family studies*. Colorado: Colorado State Univerity, 1981.

125. Bornstein M, Cote L. "Who is sitting across from me?" Immigrant mothers' knowledge of parenting and children's development. *Pediatrics* 2004;114(5):e557-e564

126. Abidin R, Wilfong E. Parenting stress and its relationship to child health care. *Children's Health Care* 1989;18(2):114-6

127. Bifulco A, Mahon J, Kwon J, Moran P, Jacobs C. The Vulnerable Attachment Style Questionnaire (VASQ): an interview-based measure of attachment styles that predict depressive disorder. *Psychol Med* 2003;33(6):1099-110

128. Bifulco A, Moran P, Ball C, Bernazzani O. Adult Attachment Style I: Its relationship to clinical depression. *Social Psychiatry and Psychiatric Epidemiology* 2002;37:50-59

129. Derogatis L. *SCL-90-R: Administration, scoring, and procedures manual (3rd ed.)*. Minneapolis: Derogatis, 1994.

130. Horvath A, Greenberg L. Development and validation of the Working Alliance Inventory. *Journal of Counseling Psychology* 1989;36:223-233

131. Guédeney N. Validation de la version française du WAI: Working Alliance Inventory. *Social psychiatry and psychiatric epidemiology* 2005;in press

# Annexe 1a : Formulaire d’information et de consentement

**Formulaire d’information et**

**de consentement pour une personne participant**

**à une recherche biomédicale**

Compétences parentales et Attachement dans la Petite Enfance - Diminution des risques liés aux troubles de santé mentale et Promotion de la résilience

**Etude CAPEDP**

**Consentement pour la mère**

**Les pages de ce document doivent être numérotées (1/nombre total de pages), paraphées par le médecin investigateur et les personnes donnant leur consentement ; la dernière page doit être datée et signée par ces mêmes personnes.**

Le docteur(1)……………….., médecin investigateur, m’a proposé de participer à la recherche biomédicale intitulée « Compétences parentales et Attachement dans la Petite Enfance - Diminution des risques liés aux troubles de santé mentale et Promotion de la résilience » (CAPEDP).

Le médecin m’a précisé que j’étais libre d’accepter ou de refuser de participer à cette recherche.

Afin d’éclairer ma décision, j’ai reçu et bien compris les informations suivantes :

| La grossesse et les deux premières années de la vie du bébé sont une période essentielle pour le développement de l’enfant. La mère a souvent besoin d’information et de soutien pour faire face aux questionnements et aux problèmes et élever son enfant – plus particulièrement si c’est son premier enfant, si elle est jeune et si elle a d’autres défis à relever en même temps. Le but de cette recherche est d’évaluer l’effet d’un programme d’aide supplémentaire pour ces mères, fait de visites régulières à domicile par des intervenantes formées aux enjeux de la petite enfance. Il s’agit dans cette recherche de comprendre de quelle manière ce programme d’aide peut favoriser la création de relations solides entre la mère et son enfant, et si ce soutien diminue le risque pour la mère de se sentir triste après la naissance, et s'il aide à consolider le développement et la santé mentale de l’enfant. 440 jeunes femmes enceintes de leur premier enfant, toutes habitant Paris et la petite couronne, participeront à cette étude. Elles entreront dans l’étude avant le dernier trimestre de grossesse. Pour savoir si cette aide est utile pour le développement psychologique de l’enfant et la santé de la mère, deux groupes de mères seront comparés. Le premier groupe a accès aux soins usuels, la maternité, la PMI, les services sociaux, le personnel des haltes-garderies et des crèches et recevra six visites à domicile par l’équipe d’évaluateurs. L’autre groupe bénéficiera, en plus des soins habituels ci-dessus, du programme d’aide fait de visites à domicile : tous les 10 à 15 jours pendant les 6 premiers mois puis toutes les 3 semaines jusqu’aux 18 mois de l’enfant et 1 fois par mois entre les 18^ème^ et 24^ème^ mois de l’enfant. Lors de ces visites, l’intervenante me proposera de discuter de l’évolution de ma grossesse, puis de celle de mon enfant, des choses agréables ou moins agréables, des difficultés qui pourraient survenir. Des moments plus informatifs pourront également m’être proposés. Les visiteurs sont des psychologues, femmes, formées spécialement à ce type d’intervention, qui travaillent avec les spécialistes de la petite enfance  C’est un tirage au sort qui décidera dans quel groupe je serai (soins comme d’habitude ou programme d’aide).  Dans les deux groupes, chaque femme aura une visite à domicile vers le 7^e^ mois de grossesse, un appel téléphonique après l’accouchement, puis d’autres visites à domicile aux 3^e^, 6^e^, 12^e^, 18^e^ et 24^e^ mois de l’enfant pour faire des observations et répondre à des questionnaires sur sa santé et celle de son enfant. Chaque visite durera de 60 à 90 minutes environ. Les évaluateurs ne connaîtront pas le groupe dans lequel j’ai été tiré au sort. Je m’engage à ne pas lui révéler.  Bien évidemment, je pourrai interrompre les visites si je le souhaite.  En tout, l’étude durera pour moi 32 mois maximum.  Il n’y a pas de prise de sang ni d’examen physique (en dehors de ceux habituellement pratiqués), ni pour moi ni pour mon enfant. |
| --- |

L'Assistance Publique - Hôpitaux de Paris, promoteur de cette recherche biomédicale a contracté une assurance conformément aux dispositions législatives, garantissant sa responsabilité civile et celle de tout intervenant auprès de la compagnie Gerling  France, 111 rue de Longchamp, 75116 Paris, par l’intermédiaire de BIOMEDIC INSURE (02 97 69 19 19) courtier en assurances.

Cette recherche a reçu l’avis favorable du Comité Consultatif de Protection des Personnes participant à une Recherche Biomédicale de Paris Saint-Louis, le ……………………

Dans le cadre de la recherche biomédicale à laquelle l’Assistance Publique – Hôpitaux de Paris me propose de participer, un traitement informatique de mes données personnelles et de celles de mon enfant va être mis en oeuvre pour permettre d’analyser les résultats de la recherche au regard de l’objectif de cette dernière qui m’a été présenté.

A cette fin, les données médicales me concernant et les données relatives à mes habitudes de vie ainsi que, mes origines ethniques ou des données relatives à ma vie personnelle ainsi que les données concernant mon enfant qui sont nécessaires à la recherche seront transmises au promoteur de la recherche ou aux personnes ou sociétés agissant pour son compte, en France ou à l’étranger. Ces données seront identifiées par un numéro de code et mes initiales. Ces données pourront également, dans des conditions assurant leur confidentialité, être transmises aux autorités de santé françaises ou étrangères, à d’autres entités de l’Assistance Publique – Hôpitaux de Paris. Conformément aux dispositions de la loi relative à l’informatique aux fichiers et aux libertés, je dispose d’un droit d’accès et de rectification. Je dispose également d’un droit d’opposition à la transmission des données couvertes par le secret professionnel susceptibles d’être utilisées dans le cadre de cette recherche et d’être traitées.

Je peux également accéder directement ou par l’intermédiaire d’un médecin de mon choix à l’ensemble de mes données médicales en application des dispositions de l’article L. 1111-7 du code de la santé publique.

Ces droits s’exercent auprès du médecin qui me suit dans le cadre de la recherche et qui connaît mon identité.

Le fichier informatique utilisé pour la recherche a fait l’objet d’une autorisation auprès de la Commission Nationale de l’Informatique et des Libertés en application des articles 40-1 et suivants de la loi “ informatique et libertés ”.

J’ai bien noté que pour pouvoir participer à cette recherche je dois être affiliée à ou bénéficier d’un régime de sécurité sociale. Je confirme que c’est bien le cas.

Après en avoir discuté et avoir obtenu réponse à toutes mes questions, j’accepte librement et volontairement de participer à la recherche décrite ci-dessus. Je suis parfaitement consciente que je peux retirer à tout moment mon consentement à participer à cette recherche et cela quelles que soient mes raisons et sans supporter aucune responsabilité. Le fait de ne plus participer à cette recherche ne portera pas atteinte à mes relations avec le médecin investigateur qui me proposera, si je le souhaite et si besoin, une autre prise en charge.

Je pourrai à tout moment demander toute information complémentaire au Dr. Antoine GUEDENEY (n° de téléphone : 01 42 55 03 09).

Si je le souhaite, à son terme, je serai informée par l’investigateur qui recueille mon consentement des résultats globaux de cette recherche.

Mon consentement ne décharge en rien l’investigateur et le promoteur de l’ensemble de leurs responsabilités et je conserve tous mes droits garantis par la loi.

L’investigateur : Personne donnant le consentement :

Fait à …………… le…………………… Fait à ……………………, le ……………………….

Nom, prénom : Nom, prénom :

Signature Signature

Ce document est à réaliser en 3 exemplaires originaux, dont l’un doit être gardé 15 ans par l’investigateur, un autre remis à chaque personne donnant son consentement et le dernier transmis au promoteur.

# Annexe 1b : Formulaire d’information et de consentement 2

**Formulaire d’information et**

**de consentement pour une personne participant**

**à une recherche biomédicale**

Compétences parentales et Attachement dans la Petite Enfance - Diminution des risques liés aux troubles de santé mentale et Promotion de la résilience

**Etude CAPEDP**

**Pour l’enfant**

**Les pages de ce document doivent être numérotées (1/nombre total de pages), paraphées par le médecin investigateur et les personnes donnant leur consentement ; la dernière page doit être datée et signée par ces mêmes personnes.**

Le docteur(1)……………….., médecin investigateur nous a proposé que notre enfant (Nom, Prénom) .................... ............... participe à la recherche biomédicale intitulée “  Compétences parentales et Attachement dans la Petite Enfance - Diminution des risques liés aux troubles de santé mentale et Promotion de la résilience  ”.

Le médecin nous a précisé que nous étions libres d’accepter ou de refuser qu’il participe à cette recherche.

Afin d’éclairer notre décision, nous avons reçu et bien compris les informations suivantes :

La grossesse et les deux premières années de la vie du bébé sont une période essentielle pour le développement de l’enfant. La mère a souvent besoin d’information et de soutien pour faire face aux questionnements et aux problèmes et élever son enfant – plus particulièrement si c’est son premier enfant, si elle est jeune et si elle a d’autres défis à relever en même temps. Le but de cette recherche est d’évaluer l’effet d’un programme d’aide supplémentaire pour ces mères, fait de visites régulières à domicile par des intervenantes formées aux enjeux de la petite enfance. Il s’agit dans cette recherche de comprendre de quelle manière ce programme d’aide peut favoriser la création de relations solides entre la mère et son enfant, et si ce soutien diminue le risque pour la mère de se sentir triste après la naissance, et s'il aide à consolider le développement et la santé mentale de l’enfant. 440 jeunes femmes enceintes de leur premier enfant, toutes habitant Paris et la petite couronne, participeront à cette étude.

Les femmes enceintes sont entrées dans l’étude avant le dernier trimestre de grossesse. Pour savoir si cette aide est utile pour le développement psychologique de l’enfant et la santé de la mère, deux groupes de mères sont comparées.

Le premier groupe a accès aux soins usuels, la maternité, la PMI, les services sociaux, le personnel des haltes-garderies et des crèches et recevra six visites à domicile par l’équipe d’évaluateurs. . L’autre groupe bénéficiera, en plus des soins habituels ci-dessus, du programme d’aide fait de visites à domicile : tous les 10 à 15 jours pendant les 3 premiers mois de l’enfant puis toutes les 3 semaines jusqu’aux 18 mois de l’enfant et 1 fois par mois entre les 18^ème^ et 24^ème^ mois de l’enfant. Lors de ces visites, l’intervenante me proposera de discuter de l’évolution de ma grossesse, puis de celle de mon enfant, des choses agréables ou moins agréables, des difficultés qui pourraient survenir. Des moments plus informatifs pourront également m’être proposés. Les visiteurs sont des psychologues, femmes, formées spécialement à ce type d’intervention, qui travaillent avec les spécialistes de la petite enfance

C’est un tirage au sort qui a décidé dans quel groupe nous serons (soins comme d’habitude ou programme d’aide).

Dans les deux groupes, chaque femme a eu une visite à domicile vers le 7^e^ mois de grossesse, et aura un appel téléphonique après l’accouchement, puis d’autres visites à domicile aux 3^e^, 6^e^, 12^e^, 18^e^ et 24^e^ mois de l’enfant pour faire des observations et répondre à des questionnaires sur sa santé et celle de son enfant. Chaque visite durera de 60 à 90 minutes environ.

Bien évidemment, nous pourrons interrompre les visites si nous le souhaitons.

En tout, l’étude durera 26 mois.

Il n’y a pas de prise de sang ni d’examen physique(en dehors de ceux habituellement pratiqués), ni pour nous ni pour notre enfant.

L'Assistance Publique - Hôpitaux de Paris, promoteur de cette recherche biomédicale a contracté une assurance conformément aux dispositions législatives, garantissant sa responsabilité civile et celle de tout intervenant auprès de la compagnie Gerling France, 111 rue de Longchamp, 75116 Paris, par l’intermédiaire de BIOMEDIC INSURE (02 97 69 19 19) courtier en assurances.

Cette recherche a reçu l’avis favorable du Comité Consultatif de Protection des Personnes participant à une Recherche Biomédicale de le .

Dans le cadre de la recherche biomédicale à laquelle l’Assistance Publique – Hôpitaux de Paris nous propose de participer, un traitement informatique de nos données personnelles et de celles de notre enfant va être mis en oeuvre pour permettre d’analyser les résultats de la recherche au regard de l’objectif de cette dernière qui nous a été présenté.

A cette fin, les données médicales nous concernant et les données relatives à nos habitudes de vie ainsi que nos origines ethniques ou des données relatives à notre vie personnelle ainsi que les données concernant notre enfant qui sont nécessaires à la recherche seront transmises au promoteur de la recherche ou aux personnes ou sociétés agissant pour son compte, en France ou à l’étranger. Ces données seront identifiées par un numéro de code et nos initiales. Ces données pourront également, dans des conditions assurant leur confidentialité, être transmises aux autorités de santé françaises ou étrangères, à d’autres entités de l’Assistance Publique – Hôpitaux de Paris. Conformément aux dispositions de la loi relative à l’informatique aux fichiers et aux libertés, nous disposons d’un droit d’accès et de rectification. Nous disposons également d’un droit d’opposition à la transmission des données couvertes par le secret professionnel susceptibles d’être utilisées dans le cadre de cette recherche et d’être traitées.

Nous pouvons également accéder directement ou par l’intermédiaire d’un médecin de notre choix à l’ensemble de mes données médicales en application des dispositions de l’article L. 1111-7 du code de la santé publique.

Ces droits s’exercent auprès du médecin qui nous suit dans le cadre de la recherche et qui connaît notre identité.

Le fichier informatique utilisé pour la recherche a fait l’objet d’une autorisation auprès de la Commission Nationale de l’Informatique et des Libertés en application des articles 40-1 et suivants de la loi “ informatique et libertés ”.

Après en avoir discuté et avoir obtenu réponse à toutes nos questions, nous acceptons librement et volontairement que notre enfant participe à la recherche décrite ci-dessus. Nous sommes parfaitement conscients que nous pouvons retirer à tout moment notre consentement à sa participation à cette recherche et cela quelles que soient nos raisons et sans supporter aucune responsabilité. Le fait de ne plus participer à cette recherche ne portera pas atteinte à nos relations avec le médecin investigateur qui nous proposera, si nous le souhaitons et si besoin, une autre prise en charge pour notre enfant.

Nous avons bien noté que pour pouvoir participer à cette recherche notre enfant doit bénéficier d’un régime de sécurité sociale. Nous confirmons que c’est bien le cas.

Nous pourrons à tout moment demander toute information complémentaire au Dr. Antoine GUEDENEY (n° de téléphone : 01 42 55 03 09).

Si nous le souhaitons, à son terme, nous serons informé(es) par l’investigateur qui recueille notre consentement des résultats globaux de cette recherche.

Notre consentement ne décharge en rien l’investigateur et le promoteur de l’ensemble de leurs responsabilités et nous et notre enfant conservons tous nos droits garantis par la loi.

L’investigateur :

Nom, prénom :

Fait à , le :  Signature

Signature des titulaires de l’exercice de l’autorité parentale :

Fait à , le :  Fait à , le :

Nom, prénom : Nom, prénom :

Qualité Qualité

Signature Signature

1. Indiquer le nom, prénom, adresse, téléphone.

**Ce document est à réaliser en 3 exemplaires originaux, dont l’un doit être gardé 15 ans par l’investigateur, un autre remis à la personne donnant son consentement et le troisième transmis au promoteur.**

# Annexe 1c : Formulaire d’information et de consentement 3

**Formulaire d’information et de consentement pour une personne participant à une recherche biomédicale**

**Etude CAPEDP :** Compétences parentales et Attachement dans la Petite Enfance - Diminution des risques liés aux troubles de santé mentale et Promotion de la résilience – **Etude ancillaire : ATTACHEMENT MERE-ENFANT**

**Les pages de ce document doivent être numérotées (1/nombre total de pages), paraphées par le médecin investigateur et les personnes donnant leur consentement ; la dernière page doit être datée et signée par ces mêmes personnes.**

Nous participe à la recherche biomédicale intitulée "Compétences parentales et Attachement dans la Petite Enfance - Diminution des risques liés aux troubles de santé mentale et Promotion de la résilience (CAPEDP)", qui évalue l'impact d'un programme d'aide supplémentaire pour les jeunes mères, constitué de visites régulières à domicile par des intervenantes formées aux enjeux de la petite enfance.

Le docteur(1)……………….., médecin investigateur, m’a proposé de participer, avec notre enfant, à une recherche d'évaluation biomédicale supplémentaire intitulée « Compétences parentales et Attachement dans la Petite Enfance - Diminution des risques liés aux troubles de santé mentale et Promotion de la résilience **Etude ancillaire Attachement Mère-Enfant** ».

Le médecin nous a précisé que j’étais libre d’accepter ou de refuser que notre enfant et nous-même participions à cette recherche.

Afin d’éclairer ma décision, nous avons reçu et bien compris les informations suivantes :

| Entre le 12^e^ et le 15^e^ mois, cent femmes avec leur enfant, sélectionnés au hasard parmi les 440 femmes de l’étude principale, participeront si elles le souhaitent, à un entretien consistant en une évaluation supplémentaires nécessitant de se déplacer à l’Université de Bichat, à la Porte de Saint-Ouen. Nous avons été sélectionnés pour être invités, avec notre enfant, à participer à cette évaluation supplémentaire. Elle est composée d'une introduction et de courts épisodes (de 3 minutes) de séparations brèves de l’enfant de sa mère ainsi que de mise en situation avec une personne inconnue de lui. Il s’agit d’observer comme l'enfant s'adapte à ces différentes situations.  Il n’y a pas de prise de sang ni d’examen physique, ni pour nous ni pour notre enfant. Tout frais lié à ce déplacement nous sera remboursé.  L’évaluation en question dure environ soixante minutes. Bien évidemment, nous pourrons interrompre cette évaluation si nous le souhaitons. |
| --- |

L'Assistance Publique - Hôpitaux de Paris, promoteur de cette recherche biomédicale a contracté une assurance conformément aux dispositions législatives, garantissant sa responsabilité civile et celle de tout intervenant auprès de la compagnie Gerling  France, 111 rue de Longchamp, 75116 Paris, par l’intermédiaire de BIOMEDIC INSURE (02 97 69 19 19) courtier en assurances.

Cette recherche a reçu l’avis favorable du Comité Consultatif de Protection des Personnes participant à une Recherche Biomédicale de Paris Saint-Louis, le ……………………

Dans le cadre de la recherche biomédicale à laquelle l’Assistance Publique – Hôpitaux de Paris nous propose de participer, un traitement informatique de nos données personnelles et de celles de notre enfant va être mis en oeuvre pour permettre d’analyser les résultats de la recherche au regard de l’objectif de cette dernière qui nous a été présenté.

A cette fin, les données médicales nous concernant et les données relatives à nos habitudes de vie ainsi que nos origines ethniques ou des données relatives à notre vie personnelle ainsi que les données concernant notre enfant qui sont nécessaires à la recherche seront transmises au promoteur de la recherche ou aux personnes ou sociétés agissant pour son compte, en France ou à l’étranger. Ces données seront identifiées par un numéro de code et nos initiales. Ces données pourront également, dans des conditions assurant leur confidentialité, être transmises aux autorités de santé françaises ou étrangères, à d’autres entités de l’Assistance Publique – Hôpitaux de Paris. Conformément aux dispositions de la loi relative à l’informatique aux fichiers et aux libertés, nous disposons d’un droit d’accès et de rectification. Nous disposons également d’un droit d’opposition à la transmission des données couvertes par le secret professionnel susceptibles d’être utilisées dans le cadre de cette recherche et d’être traitées.

Nous pouvons également accéder directement ou par l’intermédiaire d’un médecin de notre choix à l’ensemble de mes données médicales en application des dispositions de l’article L. 1111-7 du code de la santé publique.

Ces droits s’exercent auprès du médecin qui nous suit dans le cadre de la recherche et qui connaît notre identité.

Le fichier informatique utilisé pour la recherche a fait l’objet d’une autorisation auprès de la Commission Nationale de l’Informatique et des Libertés en application des articles 40-1 et suivants de la loi “ informatique et libertés ”.

Nous avons bien noté que pour pouvoir participer à cette recherche nous devons être affiliés à ou bénéficier d’un régime de sécurité sociale. Nous confirmons que c’est bien le cas.

Après en avoir discuté et avoir obtenu réponse à toutes mes questions, nous acceptons librement et volontairement de participer à la recherche décrite ci-dessus. Nous sommes parfaitement conscients que nous pouvons retirer à tout moment mon consentement à participer à cette recherche et cela quelles que soient nos raisons et sans supporter aucune responsabilité. Le fait de ne plus participer à cette recherche ne portera pas atteinte à nos relations avec le médecin investigateur qui nous proposera, si nous le souhaitons et si besoin, une autre prise en charge.

Nous pourrons à tout moment demander toute information complémentaire au Dr. Antoine GUEDENEY (n° de téléphone : 01 42 55 03 09).

Si nous le souhaitons, à son terme, nous serons informés par l’investigateur qui recueille mon consentement des résultats globaux de cette recherche.

Notre consentement ne décharge en rien l’investigateur et le promoteur de l’ensemble de leurs responsabilités et nous conservons tous nos droits garantis par la loi.

L’investigateur :

Fait à …………… le……………………

Nom, prénom :

Signature

Signature des titulaires de l’exercice de l’autorité parentale :

Fait à , le :  Fait à , le :

Nom, prénom : Nom, prénom :

Qualité Qualité

Signature Signature

Ce document est à réaliser en 3 exemplaires originaux, dont l’un doit être gardé 15 ans par l’investigateur, un autre remis à chaque personne donnant son consentement et le dernier transmis au promoteur.

# Annexe 1d : Formulaire d’information et de consentement 4

**Formulaire d’information et**

**de consentement pour une personne participant**

**à une recherche biomédicale**

Compétences parentales et Attachement dans la Petite Enfance -
Diminution des risques liés aux troubles de santé mentale et Promotion de la résilience

Etude CAPEDP

Pour la femme mère mineure

**Les pages de ce document doivent être numérotées (1/nombre total de pages), paraphées par le médecin investigateur et les personnes donnant leur consentement ; la dernière page doit être datée et signée par ces mêmes personnes.**

Le docteur(1)……………….., médecin investigateur nous a proposé que notre enfant (Nom, Prénom) ................................... participe à la recherche biomédicale intitulée “  Compétences parentales et Attachement dans la Petite Enfance - Diminution des risques liés aux troubles de santé mentale et Promotion de la résilience  ”.

Le médecin nous a précisé que nous étions libres d’accepter ou de refuser qu’il participe à cette recherche.

Afin d’éclairer notre décision, nous avons reçu et bien compris les informations suivantes :

La grossesse et les deux premières années de la vie du bébé sont une période essentielle pour le développement de l’enfant. La mère a souvent besoin d’information et de soutien pour faire face aux questionnements et aux problèmes et élever son enfant – plus particulièrement si c’est son premier enfant, si elle est jeune et si elle a d’autres défis à relever en même temps. Le but de cette recherche est d’évaluer l’effet d’un programme d’aide supplémentaire pour ces mères, fait de visites régulières à domicile par des intervenantes formées aux enjeux de la petite enfance. Il s’agit dans cette recherche de comprendre de quelle manière ce programme d’aide peut favoriser la création de relations solides entre la mère et son enfant, et si ce soutien diminue le risque pour la mère de se sentir triste après la naissance, et s'il aide à consolider le développement et la santé mentale de l’enfant. 440 jeunes femmes enceintes de leur premier enfant, toutes habitant Paris et la petite couronne, participeront à cette étude. Elles entreront dans l’étude avant le dernier trimestre de grossesse. Pour savoir si cette aide est utile pour le développement psychologique de l’enfant et la santé de la mère, deux groupes de mères seront comparés. Le premier groupe a accès aux soins usuels, la maternité, la PMI, les services sociaux, le personnel des haltes-garderies et des crèches et recevra six visites à domicile par l’équipe d’évaluateurs. . L’autre groupe bénéficiera, en plus des soins habituels ci-dessus, du programme d’aide fait de visites à domicile : tous les 10 à 15 jours pendant les 6 premiers mois puis toutes les 3 semaines jusqu’aux 18 mois de l’enfant et 1 fois par mois entre les 18^ème^ et 24^ème^ mois de l’enfant. Lors de ces visites, l’intervenante proposera de discuter de l’évolution de la grossesse, puis de celle de l’enfant, des choses agréables ou moins agréables, des difficultés qui pourraient survenir. Des moments plus informatifs pourront également être proposés. Les visiteurs sont des psychologues, femmes, formées spécialement à ce type d’intervention, qui travaillent avec les spécialistes de la petite enfance

C’est un tirage au sort qui décidera de l’attribution du groupe (soins comme d’habitude ou programme d’aide).

Dans les deux groupes, chaque femme aura une visite à domicile vers le 7^e^ mois de grossesse, un appel téléphonique après l’accouchement, puis d’autres visites à domicile aux 3^e^, 6^e^, 12^e^, 18^e^ et 24^e^ mois de l’enfant pour faire des observations et répondre à des questionnaires sur sa santé et celle de son enfant. Chaque visite durera de 60 à 90 minutes environ. Les évaluateurs ne connaîtront pas le groupe dans lequel le femme enceinte aura été tirée au sort. Celle-ci s’engage à ne pas lui révéler.

Bien évidemment les visites pourront être interrompues..

En tout, le durée de participation à l’étude sera de 32 mois maximum.

Il n’y a pas de prise de sang ni d’examen physique (en dehors de ceux habituellement pratiqués), ni pour la mère, ni i pour l’enfant.

L'Assistance Publique - Hôpitaux de Paris, promoteur de cette recherche biomédicale a contracté une assurance conformément aux dispositions législatives, garantissant sa responsabilité civile et celle de tout intervenant auprès de la compagnie Gerling France, 111 rue de Longchamp, 75116 Paris, par l’intermédiaire de BIOMEDIC INSURE (02 97 69 19 19) courtier en assurances.

Cette recherche a reçu l’avis favorable du Comité Consultatif de Protection des Personnes participant à une Recherche Biomédicale de le .

Dans le cadre de la recherche biomédicale à laquelle l’Assistance Publique – Hôpitaux de Paris me propose de participer, un traitement informatique de mes données personnelles et de celles de mon enfant va être mis en oeuvre pour permettre d’analyser les résultats de la recherche au regard de l’objectif de cette dernière qui m’a été présenté.

A cette fin, les données médicales me concernant et les données relatives à mes habitudes de vie ainsi que, mes origines ethniques ou des données relatives à ma vie personnelle ainsi que les données concernant mon enfant qui sont nécessaires à la recherche seront transmises au promoteur de la recherche ou aux personnes ou sociétés agissant pour son compte, en France ou à l’étranger. Ces données seront identifiées par un numéro de code et mes initiales. Ces données pourront également, dans des conditions assurant leur confidentialité, être transmises aux autorités de santé françaises ou étrangères, à d’autres entités de l’Assistance Publique – Hôpitaux de Paris. Conformément aux dispositions de la loi relative à l’informatique aux fichiers et aux libertés, je dispose d’un droit d’accès et de rectification. Je dispose également d’un droit d’opposition à la transmission des données couvertes par le secret professionnel susceptibles d’être utilisées dans le cadre de cette recherche et d’être traitées.

Je peux également accéder directement ou par l’intermédiaire d’un médecin de mon choix à l’ensemble de mes données médicales en application des dispositions de l’article L. 1111-7 du code de la santé publique.

Ces droits s’exercent auprès du médecin qui me suit dans le cadre de la recherche et qui connaît mon identité.

Le fichier informatique utilisé pour la recherche a fait l’objet d’une autorisation auprès de la Commission Nationale de l’Informatique et des Libertés en application des articles 40-1 et suivants de la loi “ informatique et libertés ”.

Après en avoir discuté et avoir obtenu réponse à toutes nos questions, nous acceptons librement et volontairement que notre enfant participe à la recherche décrite ci-dessus. Nous sommes parfaitement conscients que nous pouvons retirer à tout moment notre consentement à sa participation à cette recherche et cela quelles que soient nos raisons et sans supporter aucune responsabilité. Le fait de ne plus participer à cette recherche ne portera pas atteinte à nos relations avec le médecin investigateur qui nous proposera, si nous le souhaitons et si besoin, une autre prise en charge pour notre enfant.

Nous avons bien noté que pour pouvoir participer à cette recherche notre enfant doit bénéficier d’un régime de sécurité sociale. Nous confirmons que c’est bien le cas.

Nous pourrons à tout moment demander toute information complémentaire au Dr. Antoine GUEDENEY (n° de téléphone : 01 42 55 03 09).

Si nous le souhaitons, à son terme, nous serons informé(es) par l’investigateur qui recueille notre consentement des résultats globaux de cette recherche.

Notre consentement ne décharge en rien l’investigateur et le promoteur de l’ensemble de leurs responsabilités et nous et notre enfant conservons tous nos droits garantis par la loi.

L’investigateur :

Nom, prénom :

Fait à , le :  Signature

Signature des titulaires de l’exercice de l’autorité parentale :

Fait à , le :  Fait à , le :

Nom, prénom : Nom, prénom :

Qualité Qualité

Signature Signature

**Le consentement de l’enfant sera recherché Il ne peut être passé outre à son refus ou à la révocation de son consentement.**

L’enfant :

Nom, prénom :

Signature

1. Indiquer le nom, prénom, adresse, téléphone.

**Ce document est à réaliser en 3 exemplaires originaux, dont l’un doit être gardé 15 ans par l’investigateur, un autre remis à la personne donnant son consentement et le troisième transmis au promoteur.**

# Annexe 2 : Carte provisoire de la zone d’étude


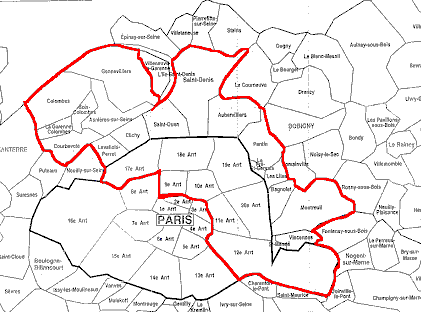


# Annexe 3 : Chronologie des visites

# Annexe 4 : Liste des centres participants à l’étude, des investigateurs et des superviseurs.

##### Hôpital Bichat-Claude-Bernard

- Pr Antoine Guédeney, (superviseur)
- Dr Romain Dugravier, (superviseur)
- Pr Patrick Madelenat,
- Pr Dominique Mahieu-Caputo,
- Dr Agnès Batallan,

##### Hôpital Louis-Mourier

- Pr Laurent Mandelbrot,
- Dr Catherine Crenn-Hebert

##### Hôpital Robert-Debré

- Pr Jean-François Oury,
- Dr Dominique Luton,
- Dr Diane Purper-Ouakil (superviseur)
- Dr Olivia Ferraud

##### Hôpital de la Pitié-Salpêtrière

- Pr Marc Dommergues,
- Dr Romain Guilherme,

##### Hôpital Saint-Antoine

- Pr Jacques Milliez,
- Dr Bruno Carbonne,

##### Hôpital Beaujon

- Pr Michel Levardon,
- Dr Carine Yver,

##### Hôpital Ville-Evrard

- Dr Bertrand Welniarz (superviseur)

##### Hôpital Maison-Blanche

- Dr Alain Haddad (superviseur)
- M. Thomas Saïas (superviseur)

1. La proximité du jeune enfant vis-à-vis de sa mère favorise son sentiment de sécurité. En effet, la sensibilité maternelle aux besoins du bébé permet à ce dernier de développer des attentes positives vis-à-vis de la relation, l’aide arrive quand elle est nécessaire, et permet de mettre les émotions en forme, jugulant ainsi le recours aux comportements violents. [↑](#footnote-ref-2)
2. Fréquence des VAD dans les études principales similaires :

   - Elmira : une fois par semaine dans le mois suivant l’inclusion (grossesse). Puis tous les 15 jours jusqu’à l’accouchement. ; en moyenne 24 en post natal (Olds, 1990)
   - Memphis : une moyenne de 7 visites durant la grossesse (0-14) puis 26 (0-71) durant les deux premières années de l’enfant (Kitzman, 1997)
   - Aronen : groupe contrôle visité 3-6 fois durant les 6 premiers mois de vie de l’enfant (entretiens informatifs). Groupe intervention : 10 fois par an durant 5 ans (40 familles par intervenante) (Aronen, 1993)
   - Barth : moyenne de 11 visites sur 6 mois (Barth, 1991)
   - Margolis : 2 à 4 visites par mois durant la première année de l’enfant par une infirmière (Margolis, 2001)
   - Denver : moyenne de 6,3 visites (0-21) durant la grossesse et 21 (0-71) visites durant les deux premières années de l’enfant (Olds, 2004)

   [↑](#footnote-ref-3)
3. En moyenne [↑](#footnote-ref-4)
4. Les psychologues-intervenants seront systématiquement appelés « intervenants » dans ce document. L’objectif de l’intervenant n’est pas, dans le cadre de cette recherche-action de mener des consultations psychologiques à domicile. La formation (ainsi que la supervision) s’attacheront ainsi à définir un profil de poste particulier (car aujourd’hui inexistant dans ce contexte), celui d’*intervenant communautaire à domicile*, poste pour lequel une formation initiale en santé mentale est essentielle. [↑](#footnote-ref-5)
5. Selon Carl Rogers :

   - L'élément vivant est la relation dynamique entre les personnes,

   - L'interrelation doit aboutir, pour le sujet, à une assistance contribuant à un meilleur épanouissement,

   - La relation telle qu’elle est instaurée représente la construction d'un nouvel environnement pour le sujet. Elle lui permet de chercher plus efficacement la solution à ses problèmes, sa solution,

   L'entretien est le moyen par lequel le sujet est amené à poser son problème et par lequel on l'amène à y voir plus clair. Ainsi la relation qui se développe est spécifique et diffère des autres genres d'interrelations humaines. Il s’agit de :

   1) Comprendre le problème tel qu’il se pose pour telle personne singulière,

   2) Aider le sujet à évoluer personnellement dans le sens d'une meilleure adaptation au contexte social, psychologique ou économique.

   La technique de l'entretien en découle. Il doit être peu directif et centré sur le sujet selon :

   a) Une attitude d'intérêt ouvert, c'est-à-dire une disponibilité intégrale, sans préjugé ni a priori d'aucune sorte, une manière d'être et de faire qui soit un encouragement continu à l'expression spontanée du sujet,

   b) Une attitude de non-directivité ; c'est à dire qu'il n'y a rien à chercher ou à vérifier, et que le sujet a l'initiative complète dans sa présentation du problème et dans son itinéraire,

   c) Une attitude de non-jugement permettant de tout recevoir, de tout accueillir, sans critique ni culpabilisation outrancière de la personne,

   d) Une intention authentique de comprendre l'interlocuteur dans sa propre langue, de penser dans ses termes, de découvrir son univers, c'est-à-dire de saisir les significations que la situation a pour le sujet,

   e) Un effort continu pour rester objectif tout au long de l'entretien. [↑](#footnote-ref-6)
